# Musculoskeletal organoids-on-chip uncover muscle–bone communication under intermittent hypoxia

**DOI:** 10.1093/nsr/nwaf214

**Published:** 2025-05-27

**Authors:** Xianqin Tong, Minchao Liu, Jiao Li, Weihua Zhang, Rong Hu, Gang Yang, Jiajia Deng, Yuanyuan Li, Xiaomin Li, Yuehua Liu

**Affiliations:** Department of Orthodontics, Shanghai Stomatological Hospital & School of Stomatology, Fudan University, Shanghai 201100, China; Shanghai Key Laboratory of Craniomaxillofacial Development and Diseases, Fudan University, Shanghai 201100, China; Department of Chemistry & Laboratory of Advanced Materials, Fudan University, Shanghai 200000, China; Department of Orthodontics, Shanghai Stomatological Hospital & School of Stomatology, Fudan University, Shanghai 201100, China; Shanghai Key Laboratory of Craniomaxillofacial Development and Diseases, Fudan University, Shanghai 201100, China; Department of Orthodontics, Shanghai Stomatological Hospital & School of Stomatology, Fudan University, Shanghai 201100, China; Shanghai Key Laboratory of Craniomaxillofacial Development and Diseases, Fudan University, Shanghai 201100, China; Shanghai Key Laboratory of Craniomaxillofacial Development and Diseases, Fudan University, Shanghai 201100, China; Department of Orthodontics, Shanghai Stomatological Hospital & School of Stomatology, Fudan University, Shanghai 201100, China; Shanghai Key Laboratory of Craniomaxillofacial Development and Diseases, Fudan University, Shanghai 201100, China; Department of Orthodontics, Shanghai Stomatological Hospital & School of Stomatology, Fudan University, Shanghai 201100, China; Shanghai Key Laboratory of Craniomaxillofacial Development and Diseases, Fudan University, Shanghai 201100, China; Department of Orthodontics, Shanghai Stomatological Hospital & School of Stomatology, Fudan University, Shanghai 201100, China; Shanghai Key Laboratory of Craniomaxillofacial Development and Diseases, Fudan University, Shanghai 201100, China; Shanghai Key Laboratory of Craniomaxillofacial Development and Diseases, Fudan University, Shanghai 201100, China; Department of Chemistry & Laboratory of Advanced Materials, Fudan University, Shanghai 200000, China; Department of Orthodontics, Shanghai Stomatological Hospital & School of Stomatology, Fudan University, Shanghai 201100, China; Shanghai Key Laboratory of Craniomaxillofacial Development and Diseases, Fudan University, Shanghai 201100, China

**Keywords:** muscle–bone communication, organoids-on-chip, organoid, intermittent hypoxia, Janus mesoporous nanoparticles, mitochondria-targeted therapy

## Abstract

Muscle and bone have intimate biochemical associations spatiotemporally. Yet, the muscle–bone dynamic alterations under intermittent hypoxia (IH) remain unclear, primarily due to the lack of suitable microphysiological models. Herein, we developed a novel musculoskeletal organoids-on-chip (MSK OoC), advancing an integrated study of muscle–bone biochemical communication and personalized interventional strategies. Within this MSK OoC, muscle organoids (MOs) replicate *in vivo* micro-architecture, while bone organoids mimic both the formation and remodeling processes. Utilizing MSK OoC, we discovered that IH-induced muscle pathology suppressed osteogenesis but stimulated osteoclastogenesis. The mitochondria protein Sirt3 in muscle played a pivotal role in regulating bone metabolism via myokine Cxcl5. Besides, mitochondria-targeting sequence-mediated Sirt3 overexpression in MOs effectively reversed bone deterioration. To validate mitochondria-targeted therapeutics, a Janus silica nano-vehicle was adopted to deliver resveratrol upon MSK OoC, effectively rescuing the pathological muscle–bone dysfunction. This study highlights the potential of the MSK OoC platform for investigating interorgan communication and developing precise nanomedicine therapies.

## Teaser

This study introduces a novel musculoskeletal Organoids-on-chip to explore muscle-bone biochemical communication under intermittent hypoxia, revealing Sirt3's role in regulating bone metabolism and validating mitochondria-targeted nanomedicine therapies.

## INTRODUCTION

Muscle and bone collaborate as a unit throughout the physiological processes of development, growth and aging [1]. Clinical evidence indicates that muscle atrophy increases the risk of osteoporotic fractures, suggesting that dysfunctional muscle may disturb bone metabolism. Initially recognized as an endocrine organ in 2000, muscle interacts with bone through both mechanical stimuli and biochemical molecule networks [2]. Myokines spatiotemporally mediate the bone metabolism and, since then, were identified as proteins, transforming growth factor [3], extracellular vesicles [4], etc., yet the biochemical muscle-to-bone pathways remain largely unclear.

Skeletal muscle dysfunction and osteoporosis often coexist in diseases associated with intermittent hypoxia (IH), such as chronic obstructive pulmonary disease [5], obstructive sleep apnea [6], etc. Hypoxia-associated muscle malfunction can trigger or worsen bone-metabolism imbalance, such as decreased bone mineral density and the activation of resorption markers [5]. Understanding the muscle-to-bone communication in pathological hypoxia could aid in preventing bone complications. However, until now, a suitable patient-derived model for studying this communication has been lacking.

A traditional 2D cell culture fails to replicate native organ structure and function, while animal models involve ethical concerns and limited physiological relevance to humans. The FDA Modernization Act 2.0, passed in 2022, no longer mandates animal testing for new drugs, sparking the advancement of preclinical models [7]. Organoid technology and organoids-on-chip (OoC) engineering have pioneered the spatio-temporal construction and modeling of native microscale tissue architecture [8], enabling personalized medicine [9]. Surface-directed 3D engineered skeletal muscle organoids (MOs) have been developed to recapitulate tissue micro-architecture and function [10,11]. Bone-metabolism homeostasis coordinates continuous skeletal formation and remodeling throughout the lifetime [12]. Despite progress in the research of 3D bone organoids (BOs) [13,14], achieving dynamic bone metabolism and replicating the osteoblasts–osteoclasts interaction *in vitro* remains challenging [15,16], hindering the development of muscle–bone microphysiological systems. The musculoskeletal OoC (MSK OoC) platform may address these challenges by integrating both metabolisms into a single chip, providing a more comprehensive system through which to study muscle–bone interplay under pathological conditions [17,18], such as IH. Developing such platforms is urgently needed, yet currently lacking, to fully advance our understanding of musculoskeletal diseases and accelerate the discovery of targeted therapies.

In this study, we created an open dish-like MSK OoC to mimic the biochemical muscle–bone communication microenvironment. This platform allowed us to uncover the critical role of mitochondria-specific Sirt3 in regulating muscle–bone biochemical communication, with Sirt3 deficiency in MOs leading to impaired osteogenesis and increased osteoclastogenesis. We also discovered that irregular expression and secretion of Cxcl5 from MOs under IH interfered with osteoblasts–osteoclasts interaction, further exacerbating bone pathology. Importantly, targeted overexpression of Sirt3 in MOs mitochondria could rescue pathological muscle–bone phenotype under IH. To translate these findings into potential therapeutic applications, we employed a novel mitochondria-targeted Janus mesoporous silica and hydrophobic periodic mesoporous organosilica (MSN&PMO) vehicle to deliver resveratrol (RES), effectively reversing the detrimental effects of IH on muscle–bone communication. Overall, the MSK OoC could be a promising preclinical platform for screening potential molecule therapeutic targets and evaluating advanced nanomedicine.

## RESULTS

### Design of MSK OoC and on-chip characterization under normoxia

**Figure 1.**
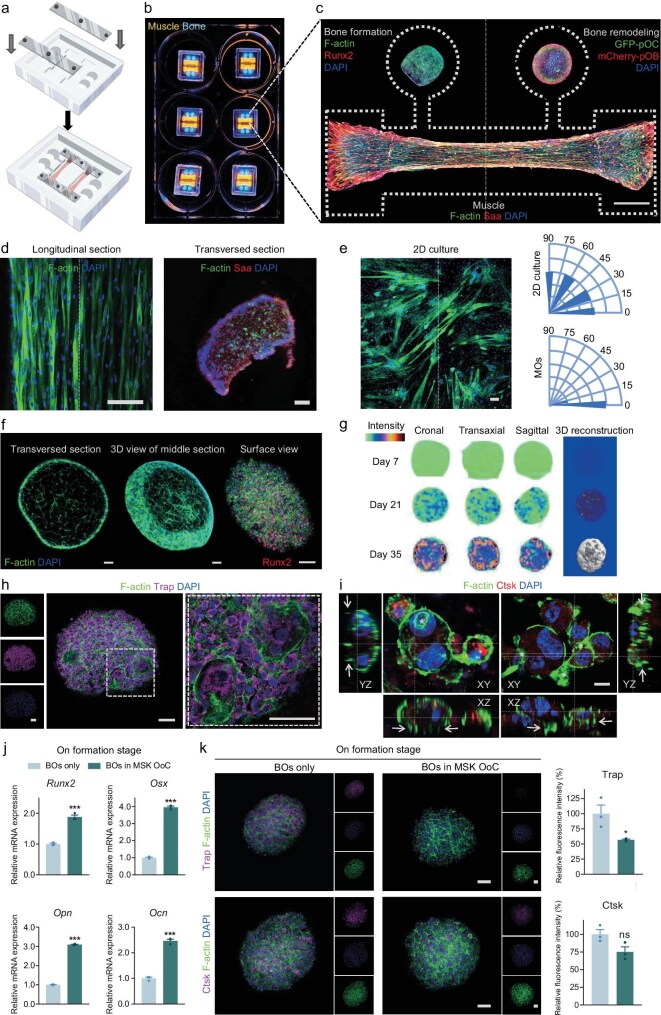
Design of the MSK OoC and on-chip characterization under normoxia. (a) Model of MSK OoC. (b) Photograph of a six-well plate with the MSK OoC in which the middle channel marked as ‘muscle’ was designed to seed MOs while side chambers marked as ‘bone’ house BOs. (c) Confocal microscopy images of MOs, bone formation and remodeling organoids in the MSK OoC (pOB: pre-osteoblast, pOC: pre-osteoclast). Scale bar, 1000 μm. (d) The transverse and longitudinal section view of MOs fabricated in an anisotropic manner and balanced distribution inside. Scale bar, 100 μm. (e) F-actin staining of myotube in 2D culture and quantitative analysis of alignment of myofiber between 2D and 3D organoid. Scale bar, 100 μm. (f) Confocal microscopy images of Runx2/F-actin staining BOs at the bone-formation stage: transverse section, 3D reconstruction view of the middle section, surface view. Scale bar, 100 μm. (g) Micro-CT scanning of mineralized BOs. (h) Confocal microscopy images of F-actin and Trap staining BOs at the bone-remodeling stage. Scale bar, 100 μm. (i) Z-stack 3D scanning reconstruction and slice view of functional osteoclasts with F-actin ring and pseudopods. Scale bar, 10 μm. Asterisk, F-actin ring; arrow, pseudopods. (j) Osteogenic mRNA expression of BOs at the formation stage of the bone-only OoC and MSK OoC under normoxia. (k) Representative confocal images and the corresponding semi-quantitative analysis of Trap and Ctsk expression of remodeling BOs on the bone-only OoC and MSK OoC under normoxia. Scale bar, 100 μm. ns, not significant; *P < 0.05; **P < 0.01; ***P < 0.001; mean ± s.e.m., *n* ≥ 3; *P* values were calculated by using two-tailed unpaired *t*-test.

Building on our previous work that focused on MOs [10], the current study advances this foundation by developing a muscle–bone interaction chip in which both MOs and BOs are integrated within the MSK OoC platform. As shown in Fig. S1a, the chip boasts a central channel accompanied by two flanking wells, in addition to two petite channel connections. The central channel was employed for the cultivation and differentiation of MOs, as in our previous work, with mechanical stabilization being guaranteed by using a silicone frame at either extremity (Fig. 1a). For the construction of BOs, the pre-osteoblasts were seeded onto another chip (Fig. S1b and c). The MOs were prepared by following a 14-day differentiation and subsequently co-cultured with the as-prepared BOs in the flanking wells on the formation and remodeling stages for varying durations as per experimental requirements, to observe alterations in their characteristic indicators (Fig. 1b and c).

MOs were fabricated on the chip and exhibited a well-organized fiber alignment along the longitudinal axis in an anisotropic manner upon the MSK OoC, accompanied by the migration of nuclei from the center to the periphery, which represented a crucial characteristic of muscle maturation. The cross-sectional slice revealed a balanced distribution of cells within MOs (Fig. 1d). In comparison, muscle fiber in 2D culture conversely displayed only a partial organization and showcased an overall state of disarray, unveiled by the quantitative analysis (Fig. 1e). The MOs in the MSK OoC displayed superior dynamic contraction efficiency with synchronized and coordinated contraction (Fig. S2a) and good cell viability (Fig. S2b).

The optimal scaffold ratio for bone formation was identified as 1:1 matrigel to collagen, which showed the best balance of cell distribution, extension and cell viability (Fig. S3a and b), along with the highest expression of key osteogenic markers such as Runt-related transcription factor 2 (Runx2) and Osterix (Osx) (Fig. S3d). At the bone-formation stage, the results showed evenly distributed and well-extended cells, with a dense layer of osteoblasts covering the surface of the organoid precursor. Runx2 immunostaining confirmed that the surface cells were thriving, with almost all of them expressing the crucial osteogenic transcription factor (Fig. 1f). The progressive messenger ribonucleic acid (mRNA) profile of osteogenic markers in BOs indicated that the osteogenic capacity would reach its peak at 21 days (Fig. S3c). To assess the mineralization capability, the osteogenic organoid precursors were induced in osteogenic induction medium for 5 weeks. To further confirm the deposition of hydroxyapatite (HA) in the extracellular matrix, Fourier transform infrared (FTIR) spectroscopy was performed on the organoid samples in comparison to an HA standard sample. The functional moieties of the BOs samples were consistent with those in the HA standard, demonstrating the active ability to deposit HA for calcification (Fig. S3e). To further observe the morphology and extent of calcification, micro-CT scanning and reconstruction were performed on the BOs at 7, 21 and 35 days (Fig. 1g). These results tell us that our construction of BOs featured a favorable proliferation of osteoblasts, along with pronounced expression of osteogenic markers, mineralization and deposition of HA.

At the remodeling stage of the BOs on the MSK OoC, we utilized lentiviral transfection to label osteogenic precursor cells and mononuclear cells with monomeric cherry fluorescent protein (mCherry) and green fluorescent protein (GFP), respectively (mCherry for pre-osteoblasts, GFP for pre-osteoclasts). This allowed us to observe osteoblast–osteoclast interaction and an optimal macrophage density of 1∼5 × 10^4^ cells was established (Fig. S4a). Characteristic multinucleated giant cells developed on the surface and exhibited expression of tartrate-resistant acid phosphatase (Trap) enzymes on the 49th day (Fig. 1h). Cathepsin K (Ctsk)-positive osteoclast exhibited pseudopods extending deeply into the mineralized matrix, as seen from the XZ and YZ views of the Z-stack 3D scanning reconstruction on the 42th day. Notably, osteoclasts on the substrates of BOs developed a comparatively thick actin ring positioned significantly away from their periphery (Fig. 1i and Fig. S4d), differently from 2D culture on the plate but consistently with rings on bone extracellular matrix-based substrates reported before [19,20]. We also presented the Trap immunohistochemistry staining of trabecular bone *in vivo* and BOs. Trap-positive osteoclast adhered to the BOs, and pseudopods toward the substrates could be observed, consistently with the bone-remodeling process on the surface of the trabecular bone (Fig. S4b and c). These results indicate that macrophages can assemble on the surface of BOs and transform into fully functional multinucleated osteoclasts, effectively replicating the *in vivo* bone-remodeling scenario.

The aforementioned data effectively validated the successful *in vitro* construction of MOs and BOs. The MSK OoC provided a platform on which to simulate the dynamic biochemical interaction between muscle and bone. To explore how muscle affects bone metabolism, BOs at the formation/remodeling stage were co-cultured with well-differentiated MOs under normoxia conditions. During the bone-formation phase, MOs were observed to significantly enhance osteogenic differentiation (Fig. 1j). However, after 2-day co-culture, muscle secretions significantly inhibited osteoclastogenesis, as evidenced by the decreased expression of Trap and Ctsk (Fig. 1k). These findings suggest that muscle may biochemically promote bone development throughout life, as indicated by the MSK OoC.

### The biochemical communication between IH-injured MOs and BOs on the MSK OoC

**Figure 2.**
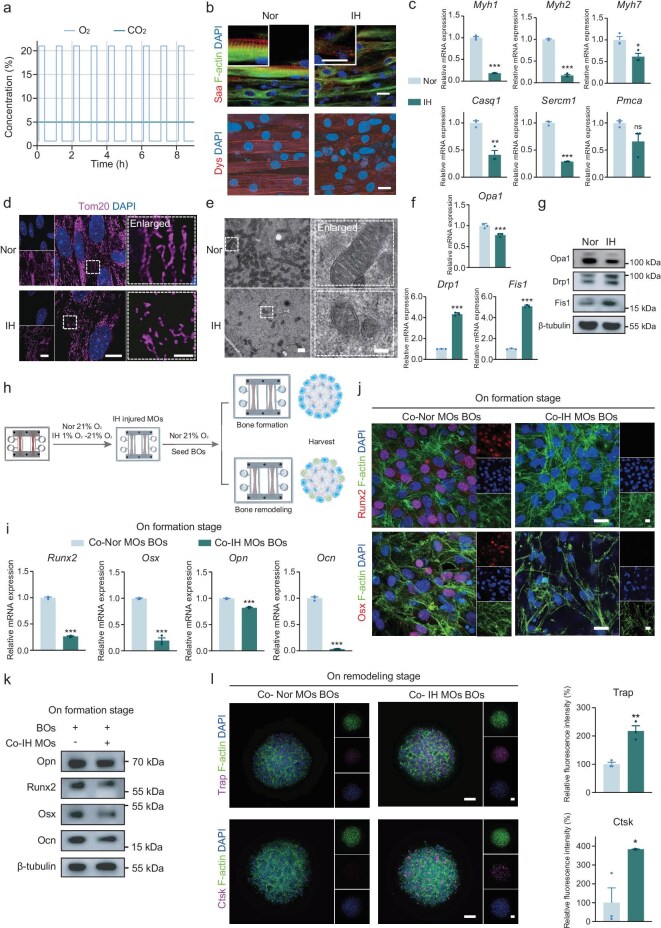
Biochemical communication between IH-injured MOs and BOs on the MSK OoC. (a) O_2_ and CO_2_ concentration during the IH cycles. (b) Representative confocal images of MOs stained with Dys and Saa under normoxia (Nor) or IH conditions. Scale bar, 20 μm. (c) mRNA expression levels related to muscle differentiation and calcium homeostasis. (d) Representative confocal images showing mitochondria net integrity in MOs after IH treatments. Scale bars, 10 μm (left, middle), 2 μm (right). (e) Representative TEM images of mitochondria in the MOs after IH treatments, illustrating mitochondrial fragmentation and mitochondria genesis. Scale bar, 400 nm. (f) mRNA expression and (g) protein expression related to mitochondria dynamics following IH treatments. (h) Schematic illustration of the BOs construction in the MSK OoC, with MOs pretreated with IH. (i) mRNA expression, (j) representative confocal images and (k) protein expression of BOs on the formation stage co-cultured with IH-pretreated MOs in MSK OoC. Scale bar, 20 μm. (l) Representative confocal images and the corresponding semi-quantitative analysis of Trap and Ctsk expression in BOs on the remodeling stage after co-culture with IH-pretreated MOs. Scale bar, 100 μm. ns, not significant; *P < 0.05; **P < 0.01; ***P < 0.001; mean ± s.e.m., *n* ≥ 3; *P* values were calculated by using two-tailed unpaired *t*-test.

The MSK OoC was cultured under IH cycles to investigate the effects of IH on muscle metabolism, function and the following muscle–bone communication. The IH cycle program alternated between 1% O_2_ + 5% CO_2_ (40 min) and 21% O_2_ + 5% CO_2_ (20 min) (Fig. 2a). After IH treatment, dystrophin (Dys) and sarcomeric alpha-actinin (Saa) staining revealed severe damage in MOs, including disrupted arrangement and compromised structural integrity (Fig. 2b). Real-time quantitative polymerase chain reaction (RT–qPCR) analysis demonstrated a notable downregulation of muscle-specific genes (*Myh1, Myh2* and *Myh7*) and genes related to calcium homeostasis (*Sercm1, Casq1* and *Pmca*) (Fig. 2c).

Furthermore, pathological alterations in mitochondrial homeostasis were observed. Mitochondria dynamics, which encompass fusion, fission, transportation and quality control, are critical for maintaining cellular function, adapting to stress and optimizing energy distribution. In the IH group, the disordered mitochondrial translocase of outer mitochondrial membrane 20 (Tom20) staining indicated compromised mitochondria network integrity (Fig. 2d). Transmission electron microscopy (TEM) analysis revealed fewer mitochondria with indistinct mitochondrial membrane structures and indications of fragmentation (Fig. 2e). The RT–qPCR results unveiled an upsurge in fission 1 (*Fis1*) and dynamin-related protein 1 (*Drp1*) expression, alongside a significant reduction in optic atrophy protein 1 (*Opa1*) levels during the 4-day IH cycles (Fig. 2f). Western blot (WB) results further supported these findings, showing downregulation of Opa1 and upregulation of Drp1 and Fis1 (Fig. 2g). In summary, these findings suggest that enhanced mitochondrial fragmentation causes dysfunctional mitochondria, leading to impaired muscle physiological functions.

Based on prior research on hypoxia muscle injury, we explored whether IH-induced MO injury could, in turn, induce abnormalities in bone metabolism. MOs were pre-exposed to IH for 2 days before being co-cultured with BOs (Fig. 2h). Significant downregulation of key markers, such as *Runx2, Osx* and osteocalcin (*Ocn*), was observed (Fig. 2i). Immunofluorescence staining further confirmed the decreased nuclear localization of Runx2 and Osx—critical osteogenic transcription factors—in osteoblasts on the surface of BOs precursors (Fig. 2j). WB analysis revealed a reduction in the protein levels of Runx2 and Osx (Fig. 2k). At the bone-remodeling stage, osteoclastogenesis was more pronounced in the IH-pretreated MOs group, showing greater fluorescence intensity compared with the normoxia group (Fig. 2l). Based on the MSK OoC, these results suggest that IH-induced muscle injury may lead to secondary disruptions in bone metabolism, characterized by inhibited osteogenic differentiation and enhanced osteoclastic activity.

### IH-induced Sirt3 downregulation and the following pathological muscle–bone communication in the MSK OoC

Reported as a nicotinamide adenine dinucleotide-dependent lysine deacetylase, Sirt3 is widely distributed in mitochondria-enriched regions, such as the brain, muscle, etc. Sirt3 primarily exists in the mitochondria matrix, orchestrating energy metabolism, homeostasis and dynamics [21].

**Figure 3.**
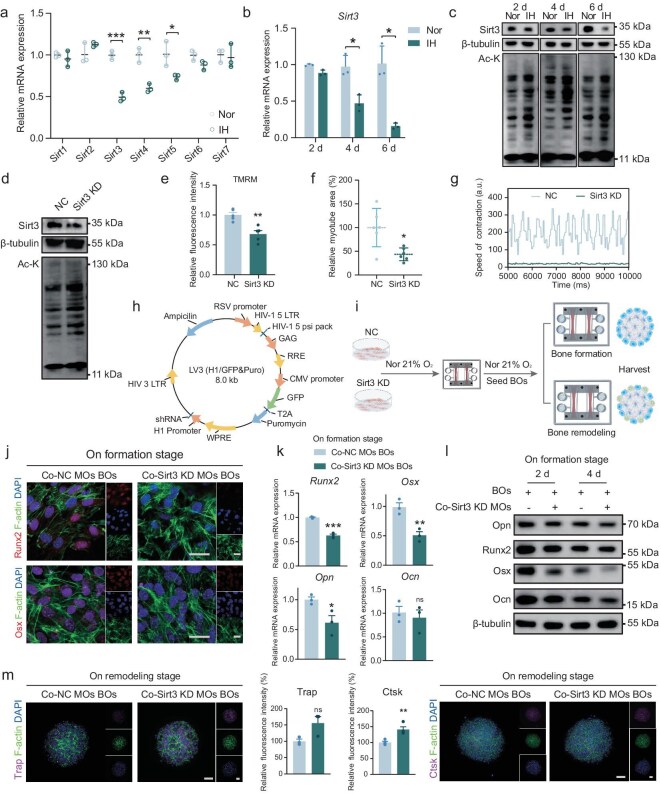
IH-induced Sirt3 downregulation and the following pathological muscle–bone communication in the MSK OoC. (a) mRNA expression of the Sirtuin family under IH. (b) mRNA and (c) protein expression of Sirt3 under nomoxia (Nor) or IH conditions. (d) Protein Ac-K level, (e) mitochondrial membrane potential, (f) myotube cross-sectional area and (g) contraction of Sirt3 KD MOs (NC: negative control; KD: knocked down). (h) Schematic diagram of LV3 lentivirus vector. (i) Schematic illustration of the BOs construction in the MSK OoC, with Sirt3 KD MOs. (j) Representative immunofluorescence staining of Runx2 and Osx, (k) mRNA and (l) protein expression of several osteoblastic markers of BOs after co-cultivation with Sirt3 KD MOs. Scale bar, 25 μm. (m) Trap and Ctsk osteoclastic enzyme activities and the corresponding semi-quantitative analysis on the remodeling stage after co-culture with Sirt3 KD MOs. Scale bar, 100 μm. ns, not significant; *P < 0.05; **P < 0.01; ***P < 0.001; mean ± s.e.m., *n* ≥ 3; *P* values were calculated by using two-tailed unpaired *t*-test (a, b, e and k) and Welch's *t*-test (f).

Based on our previous work, we noted significant changes in Sirt3 expression in the genioglossus paraffin-embedded sample of IH mice (Fig. S5a). To discover whether these changes could be replicated in MOs, we collected IH-treated MOs and conducted RT–qPCR analysis on sirtuin family members (*Sirt1-7*). Of the seven sirtuins, we found significant downregulation in *Sirt3, Sirt4* and *Sirt5*, with *Sirt3* showing the most pronounced decrease (Fig. 3a). RT–qPCR indicated a significant decrease in *Sirt3* transcription levels following IH treatment (Fig. 3b), while consistent downregulation of Sirt3 protein levels was observed in the MOs samples across three different time points. Additionally, we detected increased acetylated-lysine (Ac-K) levels of the whole protein (Fig. 3c). These data suggest that IH affects the expression and Ac-K activity of Sirt3 in MOs.

To further investigate how the mitochondrial deacetylase Sirt3 in muscle affects bone metabolism through muscle–bone communication, we constructed a Sirt3 knocked down (KD) C2C12 stable cell line by using an LV3 vector lentivirus (Fig. 3h). After puromycin selection and amplification, we generated Sirt3 KD MOs on the MSK OoC. Successful KD of *Sirt3* was confirmed (Fig. S5b), accompanied by elevated Ac-K levels of protein (Fig. 3d). Sirt3 KD MOs exhibited a reduced myotube cross-sectional area compared with the control group, a significant decrease in mitochondrial membrane potential as indicated by tetramethylrhodamine methyl ester staining as well as compromised contraction speed (Fig. 3e–g). Additionally, mitochondrial markers *Drp1* and *Fis1* were upregulated, *Opa1* was downregulated and the integrity of the mitochondria network was also compromised [22], consistently with the changes observed under IH (Fig. S5c and d).

After co-culturing with Sirt3 KD MOs, immunofluorescence staining revealed a decrease in the nuclear expression of osteogenic transcription factors Runx2 and Osx in BOs (Fig. 3i and j). WB and RT–qPCR results consistently showed that co-culture with Sirt3 KD led to a reduction in the osteogenic differentiation capacity (Fig. 3k and l). Moreover, the average fluorescence intensity of Trap- and Ctsk-positive osteoclasts on the surface of the BOs increased after co-culture, suggesting that Sirt3 KD MOs promoted osteoclast activity (Fig. 3m). Overall, muscle-specific Sirt3 plays a critical role in mediating bone metabolism within the MSK OoC. Furthermore, the MSK OoC is an ideal platform for the precise gene silencing of individual organoids, enabling detailed exploration of the dynamic interaction between muscle and bone.

### Bone-metabolism regulation by muscular Sirt3–Cxcl5 axis under IH

**Figure 4.**
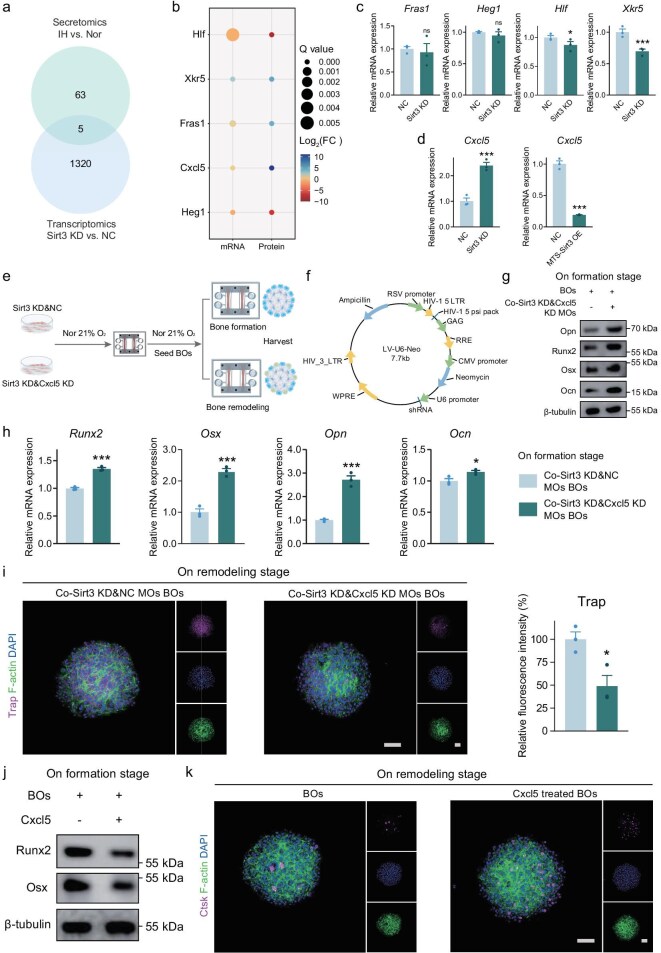
Bone-metabolism regulation by muscular Sirt3–Cxcl5 axis under IH. (a) Venn diagram presenting the overlap numbers between secretomics of IH-treated MOs and transcriptomics of Sirt3 KD MOs (KD: knocked down; NC: negative control). (b) Bubble plots showing the fold change (FC) and Q value of five overlapped genes and proteins. (c) *Fras1, Hlf, Heg1* and *Xkr5* gene expression of Sirt3 KD MOs. (d) *Cxcl5* gene expression of Sirt3 KD and OE MOs (MTS: mitochondrial targeting sequence; OE: overexpression). (e) Phenotype of BOs within the MSK OoC co-cultured with Cxcl5 KD&Sirt3 KD MOs. (f) Schematic diagram of LVU6 lentivirus vector. (g) Osteogenic protein and (h) mRNA expression of BOs co-cultured with Sirt3 KD&Cxcl5 KD MOs within MSK OoC. (i) Representative confocal images of Trap activity of BOs co-cultured with Sirt3 KD&Cxcl5 KD MOs on remodeling stage. Scale bar, 100 μm. (j) Protein expression of Runx2 and Osx of BOs on the formation stage after Cxcl5 treatment. (k) Representative confocal images of Ctsk activity of BOs after Cxcl5 treatment. Scale bar, 100 μm. ns, not significant; *P < 0.05; **P < 0.01; ***P < 0.001; mean ± s.e.m., *n* ≥ 3; *P* values were calculated by using two-tailed unpaired *t*-test.

Given that Sirt3 deficiency of MOs disrupted bone metabolism, we hypothesized that decreased Sirt3 expression could significantly affect the secretion of key proteins involved in bone metabolism, potentially playing a pivotal role in bone metabolism. To test this hypothesis, we analysed the supernatant proteins from MOs under normoxia and IH conditions by using secretomics and integrated the findings with ribonucleic acid (RNA) profiling of Sirt3 KD MOs by using transcriptomics (Fig. 4a). Five genes—Fraser extracellular matrix complex subunit 1 (*Fras1*), heart development protein with epidermal growth factor-like domains 1 (*Heg1*), hepatic leukemia factor (*Hlf*), XK-related protein 5 (*Xkr5*) and C–X–C motif chemokine ligand 5 (*Cxcl5*)—exhibited significant expression differences. Notably, of the five shared altered genes, *Cxcl5* emerged as the only secretory cytokine, showing a 1000-fold increase under the IH condition (Fig. 4b). RT–qPCR was performed on Sirt3 KD and Sirt3 overexpression (OE) MOs, confirming that Sirt3 is upstream of Cxcl5 and regulates its expression (Fig. 4c and d). In Sirt3 KD MOs, the phosphorylation of P65 was enhanced and thus the nuclear translocation of p65 was increased (Fig. S6a) [23]. When Sirt3 KD cells were treated with BAY 11–7082, which is a specific (nuclear factor kappa-B) NF-κB inhibitor (10 μM for 2 h), the *Cxcl5* mRNA level decreased by >50%, indicating that Cxcl5 might operate downstream of the NF-κB (Fig. S6b). Further analysis by using the JASPAR database identified the transcription factor core binding sequence in the Cxcl5 promotor (Fig. S6c). These findings indicated that Sirt3 in muscle mediates the expression of Cxcl5, related to the NF-κB pathway (Fig. S6d).

Cxcl5, also known as epithelial-derived neutrophil-activating protein 78 (ENA-78), is a cytokine in the CXC chemokine family [24]. It has been implicated in the development and progression of various diseases, including inflammatory diseases, metabolism-related disorders, etc. To ascertain whether Cxcl5 plays a key role in muscle–bone communication, the phenotype of BOs was evaluated when Cxcl5 was downregulated in Sirt3-deficient MOs (Fig. 4e). Sirt3 KD C2C12 cells were transfected with either a negative LVU6 vector or an effective Cxcl5 KD lentiviral LVU6 vector (Fig. 4f), which were confirmed and then used to form MOs on the MSK OoC (Fig. S7a and b). After the BOs were co-cultured with Cxcl5 KD MOs for 2 days, a recovery in gene and protein expression for osteogenic differentiation was observed (Fig. 4g and h). Additionally, bone remodeling showed a marked reduction in Trap expression (Fig. 4i). To elucidate the role of Cxcl5 in bone metabolism [25], BOs at various stages were treated with recombinant Cxcl5 protein. During the bone-formation stage, 2-day treatment with Cxcl5 led to significant inhibition of osteogenic markers such as Runx2 and Osx (Fig. 4j). Conversely, during the remodeling stage, osteoclasts exhibited increased Ctsk activity following Cxcl5 treatment (Fig. 4k). Interestingly, osteoclasts were highly sensitive to Cxcl5 stimulation in the presence of a receptor activator of the nuclear factor-κB ligand (Rankl) recombinant protein, with significantly elevated matrix metalloproteinase 9 (*Mmp9*), *Trap, Ctsk* mRNA expression (Fig. S8a). The *Rankl*/osteoclastogenesis inhibitory factor (*Opg*) ratio increased, suggesting that Cxcl5 enhances the activity of bone resorption through osteoblast–osteoclast interaction (Fig. S8b and c) [26]. These findings suggest that the sirt3-regulated Cxcl5 secretion plays a direct and essential role in the pathological progression of muscle–bone communication.

### Sirt3-mediated mito-targeted muscle repair for bone phenotype rescue

**Figure 5.**
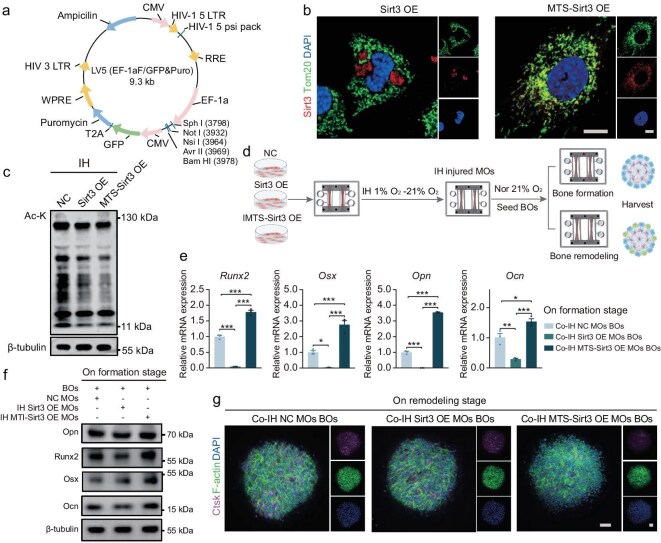
Sirt3-mediated mito-targeted muscle repair for bone phenotype rescue. (a) Schematic diagram of LV5 lentivirus vector for constructing Sirt3 OE and MTS Sirt3 OE cell lines (MTS: mitochondrial targeting sequence; OE: overexpression). (b) Representative confocal images of Sirt3/Tom20 staining of Sirt3 OE and MTS Sirt3 OE stable cell lines. Scale bar, 10 μm. (c) Protein expression of overall deacetylation within MOs under IH. (d) Schematic illustration of the BOs co-cultured with Sirt3 OE and MTS Sirt3 OE MOs in the MSK OoC. (e) Osteogenic mRNA and (f) protein expression of BOs co-cultured with Sirt3 OE and MTS Sirt3 OE MOs within the MSK OoC. (g) Ctsk osteoclastic enzyme activities at the remodeling stage co-cultured with Sirt3 OE and MTS Sirt3 OE MOs upon the MSK OoC. Scale bar, 100 μm. ns, not significant; *P < 0.05; **P < 0.01; ***P < 0.001; mean ± s.e.m., *n* ≥ 3; *P* values were calculated by using one-way ANOVA.

We further explored the potential of Sirt3 OE in MOs to rescue the bone phenotype under IH on the MSK OoC. Sirt3, a major mitochondrial deacetylase, consists of 334 amino acids, with an N-terminal mitochondrial targeting sequence (MTS), followed by 256 functional amino acids (Fig. S9) [27]. There are several splice variants of Sirt3 in murine cells, with or without N-terminal MTS. To prove that mitochondrial-targeted muscle therapy would be more effective for bone phenotype rescue, we constructed two Sirt3 isoforms through LV5 lentivirus vectors: Sirt3 OE and MTS–Sirt3 OE (Fig. 5a). Immunofluorescence showed that most Sirt3 colocalized with mitochondria in the MTS–Sirt3 OE group, whereas, in the Sirt3 OE group, only a small portion localized to mitochondria, and most of the Sirt3 dispersed in the cytoplasm, sometimes forming disordered clusters (Fig. 5b). Evaluation of the overall protein Ac-K levels under IH revealed that the MTS–Sirt3 OE group had higher deacetylation efficiency (Fig. 5c).

MOs derived from the MTS–Sirt3 OE and Sirt3 OE cell lines were then co-cultured with BOs (Fig. 5d). Under IH conditions, the MTS–Sirt3 OE group significantly increased the expression of osteogenic differentiation markers. Surprisingly, the Sirt3 OE group failed to rescue the bone phenotype and even aggravated the suppression of mRNA expression in BOs. In contrast, the MTS–Sirt3 OE group successfully reversed the bone phenotype at the formation stage (Fig. 5e and f). At the bone-remodeling stage, the Ctsk activity in the IH-injured MTS–Sirt3 OE group was efficiently decreased (Fig. 5g). These results suggest that the targeted restoration of mitochondrial protein is critical for effective muscle-to-bone phenotype rescue.

### On-chip evaluation of nanomedicine for muscle–bone phenotype rescue

**Figure 6.**
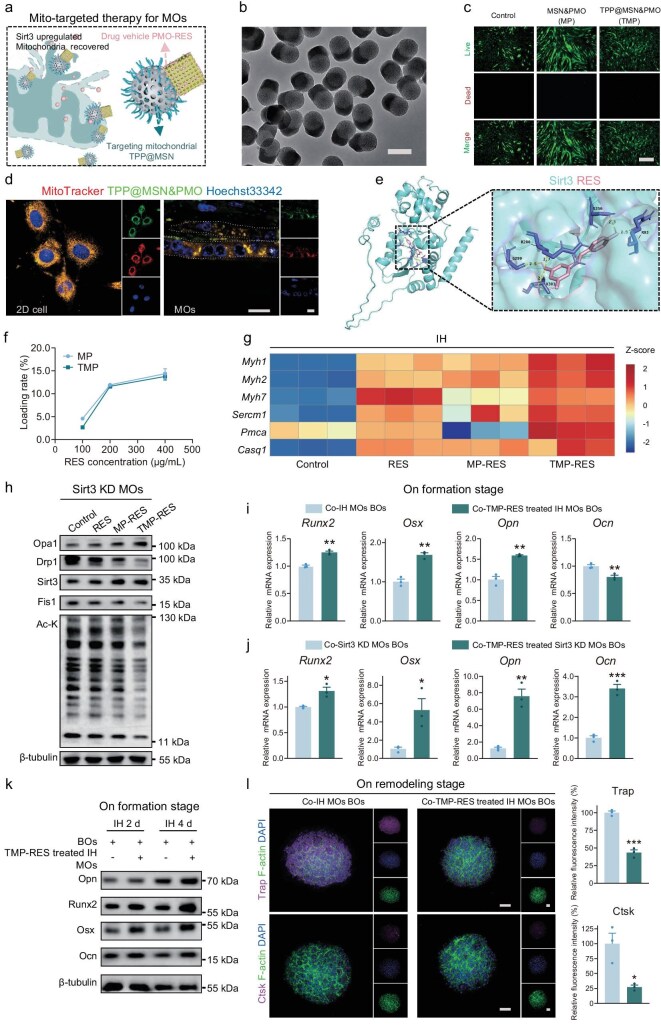
On-chip evaluation of nanomedicine for muscle–bone phenotype rescue. (a) Mitochondria-targeted delivery of resveratrol (RES) based on the TPP@MSN&PMO (TMP). (b) Representative TEM image of asymmetric MP nanoparticles. Scale bar, 200 nm. (c) Calcein/PI staining for C2C12 cells treated with TMP nano-vehicles. Scale bar, 100 μm. (d) Representative confocal images of Mito-tracker/Hoechst33342 and FITC-TPP@MSN&PMO treated C2C12 cells. Scale bar, 200 μm. (e) Molecule docking of RES bounding to Sirt3 protein. (f) HPLC analysis of saturation drug loading rate. (g) mRNA expression of muscle differentiation and contraction capacity-related genes after different treatments under IH. (h) Overall cell Ac-K and mitochondria dynamics related protein expression after different treatments within Sirt3 KD MOs upon MSK OoC. Osteogenic differentiation gene expression at the bone-formation stage co-cultured with the targeted treatment of (i) MOs under IH and (j) Sirt3 KD MOs upon the MSK OoC. (k) Osteogenic differentiation of protein expression co-cultured with targeted treatment of MOs under IH. (l) Representative confocal images and the corresponding semi-quantitative analysis of Ctsk and Trap expression of BOs at the remodeling stage co-cultured with targeted treatment of IH-injured MOs. Scale bar, 100 μm. ns, not significant; *P < 0.05; **P < 0.01; ***P < 0.001; mean ± s.e.m., *n* ≥ 3; *P* values were calculated by using two-tailed unpaired *t*-test.

Based on our findings that the mitochondria-targeted upregulation of Sirt3 was more effective at rescuing bone phenotypes, we developed an asymmetric-structured mesoporous nano platform composed of dual compartments: hydrophilic mesoporous silica (MSN) and hydrophobic periodic mesoporous organosilica (PMO) [28]. The two compartments featured pore diameters of ∼2.0 and ∼4.0 nm, respectively, with the mitochondria-targeting molecule triphenylphosphine (TPP) conjugated to the MSN subunit via amide bonds. RES has demonstrated itself as a potential therapeutic candidate for hypoxic injury in our previous research [10,29], yet its hydrophobic nature places limitations on its pharmaceutical application. RES was selectively loaded in the mesopores of the hydrophobic PMO subunit (Fig. 6a). Scanning electron microscopy and TEM images of the MSN&PMO (MP) nanocomposites show an obvious asymmetric nanostructure with a uniform size of ∼100 nm in diameter and ∼180 nm in length (Fig. 6b and Fig. S10a–c). The Brunauer–Emmett–Teller surface area was ∼914 m^2^ g^−1^, with a high pore volume of 1.17 cm^3 ^g^−1^ (Fig. S10d). The Barrett–Joyner–Halenda pore-size distribution analysis revealed two sets of mesopores at ∼2.2 and ∼3.7 nm, respectively (Fig. S10e), consistently with TEM observations. The TMP-RES (TPP@MSN&PMO-RES) exhibited good dispersion and stability, even when kept in the medium for 15 days (Fig. S10f and g). In the FTIR analysis, it is proved that the MP nanoparticles were modified with TPP through amide bonding (Fig. S10h).

Biocompatibility assessments using CCK8 confirmed that MSN&PMO (MP) and TPP@MSN&PMO (TMP) exhibited no cytotoxicity at concentrations of ≤60 μg/mL, with cell viability exceeding 90% (Fig. S11a). Calcein/PI staining of C2C12 cells showed excellent cell morphology and extension (Fig. 6c). Fluorescein Isothiocyanate (FITC)-labeled TMP (10 μg/mL) pre-treatment of C2C12 cells and MOs (24 h) demonstrated efficient mitochondrial targeting in both 2D cells and 3D organoids (Fig. 6d and Fig. S11b). The Sirt3 protein structure (ID: AF-Q8R104-F1) from the protein data bank database and the RES structure (CID: 445154) from PubChem were selected to run molecular docking (AutoDock Vina), indicating that RES bound to the Sirt3 protein with an affinity energy of –7.1 kcal/mol. Five hydrogen-bonding amino acid residues include R280, G299, V301, S256 and R93 (Fig. 6e). In addition, high performance liquid chromatography (HPLC) analysis revealed a saturated loading rate of ∼15% for the hydrophobic RES drug (Fig. 6f).

We optimized the dosage of nanomedicine based on the saturation loading rate and ultimately selected 10 μg/mL (RES loaded TMP) for subsequent experiments (Fig. S11c). Healthy MOs cultured with nanomedicine were exposed to an IH environment for 2 days. Key indicators of muscle differentiation and contraction capacity at the mRNA level showed satisfying outcomes in the targeted repair group (Fig. 6g). Compared with the drug-only and non-targeted carrier groups, the TMP-RES nanomedicine group exhibited the upregulation of Sirt3 and the most significant overall protein Ac-K modification, alongside increased Opa1 and decreased Drp1 and Fis1 levels (Fig. 6h). In addition, the nanomedicine mitigated mitochondrial fragmentation, enhanced mitochondrial network integrity (Fig. S11d) and regulated Cxcl5 expression (Fig. S11e). These data suggest that TMP-RES presents the most excellent Sirt3 activity and mitochondria repair efficiency.

To evaluate nanomedicine for muscle–bone phenotype rescue, the BOs were co-cultured with TMP-RES nanomedicine-treated IH-injured MOs and Sirt3 KD MOs. During the bone-formation stage, gene and protein expression analysis demonstrated a reduction in the inhibition of osteogenic differentiation, with a more pronounced rescue effect observed in the Sirt3 KD MSK OoC (Fig. 6i–k). During the bone-remodeling stage, there was a reduction in Ctsk and Trap enzyme activity (Fig. 6l). Additionally, we found that nanomedicine treatment led to a decrease in Rankl expression and increase in Opg, suggesting a beneficial impact on BO homeostasis (Fig. S11f). The biosafety and efficacy of the asymmetric TMP delivery system was further confirmed *in vivo* (Fig. S12a). TMP-RES administration did not cause damage to the heart, liver, spleen, lung or kidney tissue (Fig. S12b). Chronic IH resulted in muscle injury, increasing the number of centralized nuclei in the gastrocnemius muscle of the hindlimb in HE staining, while a decreasing trend was observed after nanomedicine treatment (Fig. S13a). Nanomedicine administration via intramuscular injection ameliorated the osteoporosis (Fig. S13b). These results demonstrate that the compromised muscle–bone phenotypes were successfully repaired through mitochondrial-targeted therapy, highlighting the strong link between muscle function and skeletal health, further validating the potential of the MSK OoC platform for nanomedicine screening and therapeutic evaluation.

## DISCUSSION

The biochemical interaction between muscle and bone is crucial for bone development and homeostasis, yet remains significantly underexplored. Additionally, there has been a lack of suitable platforms to comprehensively study muscle–bone communication in an integrated manner. In this study, we developed an open, dish-like chip upon our preliminary MO chip, incorporating both muscle and bone metabolic systems within the MSK OoC. Using this platform, we investigated how pathological IH disrupts the balance of bone metabolism, specifically focusing on the role of muscle-derived Sirt3. Our findings demonstrate that the muscle-specific Sirt3 plays a pivotal role in regulating both osteogenic and osteoclastic metabolism by the secretion of myokine Cxcl5. Additionally, the MSK OoC was utilized to assess the efficacy of the mitochondria-targeted nanomedicine Janus TMP-RES. Overall, the MSK OoC represents a promising platform for gaining an in-depth understanding of dynamic muscle–bone crosstalk and for making accurate preclinical predictions of nanotherapeutics.

Mechanical interdependence between muscle and bone could not entirely elucidate the phenomenon of bone loss [2,30]. Meanwhile, skeletal muscle is intricately involved in bone metabolism via myokine factors [31]. Employing OoC technology, 3D MOs and BOs are integrated into one biochemical microenvironment for research *in vitro* [32]. Muscle may exert a beneficial influence on bone development under physiological conditions [33–35]. IH interferes with muscle–bone homeostasis. Muscle mitochondrial network quality control and protein homeostasis are essential for remote organ function and systematic metabolism [36], some of which are linked to the muscle–bone axis [3,30]. On MSK OoC, our research supports that Sirt3 is downregulated in response to pathological IH [37], mediating interorgan biochemical communication via Cxcl5 between muscle and bone. More importantly, from our perspective, precise targeted therapy should be regarded as significant [38], including the expression, activity and localization of targeted proteins. The presence of MTS is related to not only the subcellular localization of Sirt3, but also the repair efficiency of bone metabolism via the muscle–bone axis. An amphiphilic Janus nanoscale vehicle [39] was thus selected for mitochondrial delivery of RES and realized optimized outcomes. Based on the MSK OoC, the strategy of mitochondrial-targeted delivery may further achieve better laboratory–clinical transition.

Despite the strengths of our MSK OoC, several limitations must be considered [40]. While our study introduces a novel protocol for investigating the interplay between muscle and bone metabolism, it is important to consider the time, cost and operational sensitivity involved in the preparation and application of the MSK OoC. Translating these laboratory findings into clinical practice poses additional challenges and further research is required to optimize the processes, scale up production and ensure rigorous quality control. The musculoskeletal system is highly complex, with diverse cell types and intricate micro-environments. While this study represents the first attempt to integrate muscle–bone biochemical crosstalk via OoC technology, there is still a long way ahead to achieve true *in vivo* simulation. Future research should focus on increasing the diversity of cell types and enhancing the complexity of the tissue structure within and between organoids. Our goal is to further unravel the intricacies of the musculoskeletal axis from developmental, physiological and pathological perspectives by replicating the mechanical/biochemical interactions between musculoskeletal elements and other characteristics in the future.

## CONCLUSION

In summary, our team have innovatively constructed a research platform that enables the observation of dynamic changes in muscle–skeletal metabolism on chips, with a particular focus on the effects of hypoxia on the muscle-to-bone signal axis. This study highlights the critical role of Sirt3 in interorgan biochemical signaling and demonstrates the potential of the MSK OoC as a powerful preclinical platform for studying muscle–bone interactions, screening molecular targets. Also, the MSK OoC presented the advantages of the amphiphilic Janus nanoscale vehicle in mitochondria-targeted delivery and suggested the potential application of evaluating advanced nanomedicine therapies. The integration of MOs and BOs within this system enables a more physiologically relevant and personalized approach to musculoskeletal disease modeling and therapeutic development.

## MATERIAL AND METHODS

The detailed material and methods can be obtained in the Supplementary Data. Drawing was partially supported by Figdraw in this article.

## Supplementary Material

nwaf214_Supplemental_File
