# Supplementary material for: Musculoskeletal organoids-on-chip uncover muscle–bone communication under intermittent hypoxia
Source: Natl Sci Rev. 2025 May 27;12(7):nwaf214. doi: 10.1093/nsr/nwaf214 (PMC12243851; doi:10.1093/nsr/nwaf214)
Supplement: nwaf214_Supplemental_File [file nwaf214_supplemental_file.pdf]

Supplementary Data for

Musculoskeletal Organoids-on-Chip Uncover Muscle-Bone Communication under Intermittent Hypoxia

Xianqin Tong[1,2,†], Minchao Liu[3,†], Jiao Li[1,2,†], Weihua Zhang[1,2], Rong Hu[2], Gang Yang[1,2], Jiajia Deng[1,2], Yuanyuan Li[1,2,*], Xiaomin Li[2,3,*] and Yuehua Liu[1,2,*]

***Corresponding authors**. E-mails: li_yuanyuan0650@fudan.edu.cn; lixm@fudan.edu.cn; liuyuehua@fudan.edu.cn
[†]Equally contributed to this work.

CONTENTS

Material and Methods

Figure S1. Size descriptions and protocols for MSK OoC fabrication.

Figure S2. Characterizations of MOs.

Figure S3. Construction and characterizations of BOs on the formation stage.

Figure S4. Construction and characterizations of BOs on the remodeling stage.

Figure S5. Sirt3 expression of muscle in vivo and characterizations of Sirt3 KD MOs.

Figure S6. NF-κB phosphorylation-induced Cxcl5 transcription in Sirt3 KD MOs.

Figure S7. Cxcl5 KD efficiency based on Sirt3 KD cell line.

Figure S8. Cxcl5 regulation of osteoblast-osteoclast interaction through Rankl/Opg.

Figure S9. Sirt3 OE and MTS-Sirt3 OE sequences.

Figure S10. Characterizations of the asymmetric-structured mesoporous.

Figure S11. Biological characterizations of the nanomedicine.

Figure S12. In vivo safety assessment of nanomedicine.

Figure S13. Evaluation of the therapeutic effectiveness of nanomedicine on muscle-bone hypoxic injury in vivo

Table 1. RT-qPCR Primers

Material and Methods

Cell culture. C2C12 mouse myoblasts and MC3T3-E1 pre-osteoblast from American Type Culture Collection (ATCC). C2C12 were cultured in DMEM containing 10% fetal bovine serum (FBS, Thermo Fisher Scientific, USA) and 1% penicillin/streptomycin (PS, Thermo Fisher Scientific, USA). MC3T3-E1 were cultured in α- MEM containing 10% FBS and 1% PS.

Mouse primary BMDMs were isolated from C57BL/6 femur and tibia bones from mice aged from 4 to 6 weeks. Briefly, bone marrow cells were flushed out using α-MEM containing 2% PS and then were filtered through a 100-μm cell strainer (Beyotime, China). Cells were cultured in α-MEM supplemented with 10% FBS and 1% PS. After 24 h, the supernatant was collected and precipitation was obtained, then BMDMs were attached in α-MEM with 10% FBS, 1% PS, and 30 ng/ml murine M-Csf (PeproTech, USA). The culture medium was replaced every other day. Cell lines and primary cells are cultured under 37 °C and 5% CO_2.

Mold and MSK OoC fabrication. Silicon mold was sculpted by CAM/CAD. The molds were put into a 6-well plate and submerged in the liquid PDMS (10:1, Momentive, USA) which were cured at 70 °C for 4 h. The PDMS soft chip was manually acquired through cutting. The middle channels were designed to seed MOs while the side circular chambers to house BOs.

Formation of engineered MOs. Use a mini needle to fix silicone strips at both ends of the middle channels on the chips. Before seeding cells, the chips were sterilized and then pre-treated using 2% pluronic-127 (Invitrogen, USA) in dd H_2O for 1h under room temperature (RT) to prevent cell adhesion. The hydrogel was formed by fibrin (10mg/mL, Sigma-Aldrich, USA), matrigel (Corning, USA), and DMEM in equal volumes of 1:1:1. Prepare 2×10^6 C2C12 cells in a centrifuge tube and gently resuspend cells using growth medium of 25 μL containing thrombin (Sigma-Aldrich, USA) and aprotinin (Sigma-Aldrich, USA). All of the procedures above were performed on ice to avoid gel solidification. The cell suspension was later evenly mixed with 55 μL hydrogel precursor and filled the middle channels. Gelation of cell-laden mixture occurred under 37 °C for 1 h and 400-500 μL DMEM containing 10% FBS, 1% PS, and 6-aminocaproic acid (ACA, Sigma-Aldrich, USA) of 1 mg/mL was added into the dish-like chip. All the chips were placed into 6-well plates in advance. In 2 days later, the medium was replaced with DMEM containing 2% horse serum (HS, Thermo Fisher Scientific, USA), 1% PS and ACA of 2 mg/mL.

Construction of in vitro BOs. Bone formation stage: PDMS chamber were fabricated in advance and detailed information was presented in Supplementary Fig. 1a. Collagen

precursor solution (Corning, USA) was prepared according to instruction with 10× PBS and 1 M NaOH. The formulation of the working hydrogel precursor solution was composed of collagen and matrigel in different ratios, allowing for the selection of the optimal combination. 5×10^6 MC3T3-E1 cells were collected in a centrifuge tube and evenly suspended in 100 μL hydrogel precursor solution. Next, filled the circular chamber with a cell-laden solution of 8-10 μL, approximately. Immersed the open multi-well PDMS chips with α-MEM with 10% FBS, and 1% PS after 1h gelation under 37 °C. After 2 days, BOs were carefully transferred to low-adhesive 96-well microplates. Mineralization induction continued for 5-7 weeks using the regular medium supplemented with beta-glycerophosphoric acid sodium (Sigma-Aldrich, USA), vitamin C (Sigma-Aldrich, USA), and dexamethasone (Sigma-Aldrich, USA).

Bone remodeling stage: After isolation and expansion, BMDMs were harvested using a cell scraper, collected by centrifugation, and subsequently resuspended in a regular medium supplemented with 30 ng/mL M-Csf (Peprotech, USA) at a density of 5×10^4cell. After 5-7 weeks of mineralization, primary OC precursors were cocultured with calcified BOs within low-adhesive 96-well plates on a shaker in the incubator overnight. The medium was replaced with regular medium supplemented with 30 ng/mL M-Csf and 50 ng/mL sRankl (Peprotech, USA).

MOs and BOs coculture on MSK OoC. Micro-physiological model: MOs were initially fabricated on MSK OoC and underwent 14-day differentiation. BOs on the formation stage underwent osteogenic induction for 4-7 days and BOs on the remodeling stage underwent osteoclast induction for 7-10 days. Next, BOs were separately placed into flanking wells of MSK OoC with mature MOs or not. The medium was replaced every 2 days using DMEM containing 2% HS and 2 mg/mL ACA. Samples were harvested after 2 or 4 days for evaluations.

IH treatment: After the maturation of MOs, the MSK OoC was put into the hypoxia incubator under IH cycles. Then, the BOs were put into flanking wells and cocultured in a Normoxia incubator for 2-4 days and harvested for evaluation.

Gene silence model: Lentivirus-mediated gene regulation was performed successfully. Several stable C2C12 cell lines were expended and fabricated into MOs according to the protocols above. BOs were harvested after co-cultivation on MSK OoC.

Mitochondria-targeted nanomedicine treatment model: MSK OoC was put into the hypoxia incubator for 4 days with 5 μg vehicles (in 5 μL ethanol) every OoC. According to the loading rate of 15%, the dosage of the RES group was calculated to be 0.75 μg RES (in 5 μL ethanol) and 5 μL ethanol in MSK OoC for the blank group. The BOs were put into flanking wells cocultured in the Normoxia incubator and harvested for evaluation.

Hypoxia treatment. Using a programmable Biospherix precision oxygen control system, a hypoxia incubator was connected to N_2, CO_2, and compressed gas mixtures (40% O_2, 60% N_2). The program was set to a cycle of 5% CO_2, 40 minutes at 1% O_2, and 20 minutes at 21% O_2, with software controlling real-time changes in gas composition within the hypoxia incubator. MSK OoC were placed in a 6-well plate and subjected to hypoxia treatment in the incubator.

Immunofluorescence. The organoids were fixed by 4% paraformaldehyde at 4 °C for 6-8 h. The samples were then permeabilized with 2.5% Triton X-100 for 4-6h under RT. After that, 5% goat serum PBS buffer was used to block and the organoids were incubated with primary antibody according to instructions at 4 °C overnight. The next day, secondary antibodies conjugated to Alexa Fluor 488, 594, 647 (1:500, Abcam) were used for 4-6 h under RT after washing away unbound secondary primary antibodies. DAPI was used to counter-stain the cell nucleus. The primary antibodies used in this study are listed as follows: anti-Saa (Sigma-Aldrich, A7811), anti-Ctsk (Santa Cruz Biotechnology, sc-48353), anti-Trap (Abcam, ab191406), anti-Dys (Abcam, Ab15277), anti-Runx2 (Affinity, AF5186,), anti-Osx (Affinity, DF7731), anti-Sirt3 (Santa Cruz Biotechnology, sc-365175), anti-Rankl (Affinity, AF0313,), anti-Opg (Affinity, DF6824), anti-Laminin (Abcam, ab11575), anti-Tom20 (Abclone, A19403).

Cryosection slices were acquired according to instructions. The slides were rinsed with PBS three times to remove OCT and permeabilized with 2.5% Triton X-100 for 15 min under RT. Primary antibody incubations were performed at 4 °C overnight after a 2-hour block under RT. Fluorescent secondary antibodies were used for 2 h under RT after rinse.

Images were acquired by confocal laser scanning microscope (NIKON, Japan) and analyzed by Image J software.

Micro-CT. The whole bone tissue or BOs underwent scanning utilizing SkyScan1272 (Bruker, Germany). To prevent dehydration during scanning, they were encased in non-polyvinylchloride parafilm. The scanning parameters were set as follows: image pixel size of 18.0 μm, source voltage at 60 kV, source current at 166 μA, rotation step of 0.400, Al 0.25 mm filter, and exposure time of 1400 ms.

WB. Organoids were grounded for 120 s at 4 °C and lysed by RIPA (Millipore, Billerica, MA, USA) with a cocktail of protease inhibitors (Sigma-Aldrich). Proteins are extracted and denatured, separated by 4% to 15% gradient gels (Tiangen, China). Then, the protein was transferred to 0.45 μm membranes (Millipore). Membranes were blocked with 5% skimmed milk at room temperature (RT) for 2 h and incubated with primary antibodies at 4 °C overnight. The membranes were incubated with horseradish peroxidase-conjugated

secondary antibodies for 2 h at RT the next day. Finally, the membranes were visualized with Femto Maximum ECL chemiluminescence substrates (Thermo Scientific). Bands were detected with Amersham Imager 600 (GE Healthcare, IL, USA) and analysis was performed by NIH Image J software. Given that cell morphology and extension changed in different gel environments, we selected total protein loading (Coomassie brilliant blue staining) as the internal reference in Fig. S3d. The others used regular internal references such as β-tubulin and β-actin. The primary antibodies used in this study are listed as follows: anti-β-tubulin (Aibisin, abs830032), anti-Runx2 (Affinity, AF5186,), anti-Osx (Affinity, DF7731), anti-Opn (Affinity, AF0227), anti-Ocn (Affinity, DF12303), anti-Sirt3 (Affinity, AF5135), anti-AC-K (Santa Cruz Biotechnology, sc-81623), anti-P-P65 (Affinity, AF2006), anti-T-P65 (Santa Cruz Biotechnology, sc-8008), anti-Cxcl5 (Affinity, DF9919), anti-Rankl (Affinity, AF0313), anti-Opg (Affinity, DF6824), anti-Drp1 (Affinity, DF7037), anti-Opa1 (Affinity, DF8587), and anti-Fis1 (Affinity, DF12005).

RT-qPCR. Organoids were grounded for 120 s at 4 °C. Total RNA was extracted from organoids by Trizol (Invitrogen, USA) and was converted into complementary DNA with Fasting PT Kit (TIANGEN, China). RT-qPCR was performed by using qPCR SYBR Green Master Mix (Yeasen, China) with LightCycler$^{@}$96 (Roche, Switzerland). The primer sequences were provided in Table 1, Supporting Information.

Lentivirus-mediated gene KD and gene OE. To establish stable cell lines, lentiviruses were employed for KD and OE in this project and corresponding selection antibiotics were provided by Genepharma Company (Shanghai, China). Lentiviral particles were added to prepared cells in the presence of polybrene.

Sirt3 KD C2C12 cell line construction: LV3 Lentiviral vector was selected and characterized with purinomycin (Puro) screening tag. To KD Sirt3 in C2C12, three sequences targeting Sirt3 were tried in the preliminary experiments, including 5’-ACAGCAACCTTCAGCAGTATG-3’, 5’-TGTCTGAAGCAGTACAGAAAT-3’, 5’-ACAAGAACTGCTGGATCTTAT-3’ (termed Sirt3 KD-1, 2, 3 respectively). Lentiviral particles were added to prepared cells (multiplicity of infection, MOI=50) in the presence of polybrene. After transfection, the cells were selected using Puro with 2 μg/mL and expanded. As a result, the first one was chosen for subsequent research through WB and RT-qPCR verification.

Sirt3 KD& Cxcl5 KD C2C12 cell line construction: Based on the Sirt3 KD cell line, we tried to construct a dual-gene KD cell line. LV-U6 vector was utilized and featured with a Neomycin screening tag. The transfection procedure was generally similar. G418 (1000 μg/mL) and Puro (2 μg/mL) were added at the same time to select during the expansion period. In the preliminary experiments, three sequences were performed (MOI=50),

including 5'-CGGTTCCATCTCGCCATTCAT-3', 5'-AGCTGCGTTGTGTTTGCTTAA-3', 5'-ACGGTGGAAGTCATAGCTAAA-3' (termed Cxcl5 KD-1, 2, 3). The first one was chosen through verification.

Sirt3 OE and MTS-Sirt3 OE C2C12 cell line construction: LV5 Lentiviral vector was selected to over-express Sirt3 (NM_022433.2) and MTS-Sirt3 (EU886466.1). Puro (2 μg/mL) was added to select cells successfully transfected (MOI=50).

Electrical stimulation and contraction analysis. The electrodes were linked with the commercial pulse generator (RIGOL, DG1022Z) and power amplifier (Aigtek, ATA-308) using nickel-coated copper wires and alligator clamps. MOs were placed in Tyode's solution and then stimulated by square pulses (voltage: 0-20 V cm^{-1}, frequency: 1 Hz, duration: 10 ms). The contraction movies were captured by microscopy (Leica, Germany) and analyzed by MUSCLEMOTION in Image J.

RNA-seq and LC–MS/MS. Transcriptomics: Sirt3 KD MOs were fabricated, went maturation, and harvested. Total RNA was isolated and purified using TRIzol reagent (Invitrogen, USA). RNA samples were quantified as RNA concentration>50 ng/μL, RNA integrity number>7.0, OD260/280>1.8, total RNA>1μg. Poly (A) RNA was captured by Dynabeads Oligo (dT)25-61005 (Thermo Fisher, USA) and fragmented by Magnesium RNA Fragmentation Module (NEB, cat. e6150, USA). Reverse-transcribed cDNA synthesized second-strands and other further preparations. The average insert size for the final cDNA library was 300±50 bp. 2×150 bp paired-end sequencing (PE150) on an illumina Novaseq™ 6000 was performed (LC-Bio Technology CO., China). Sequence quality was also verified using FASTP software. StringTie software was utilized to present expression levels for mRNA by calculating FPKM. Analysis was performed by R package edgeR.

Label-free proteomic: Normal MOs were treated with IH in the hypoxia incubator for 6 days. Medium was replaced every other day in MSK OoC and the supernatant was collected from 10-15 chips. The DDA label-free MS/MS data was analyzed by MaxQuant software (version 2.1.4.0). Contaminated protein will be removed (FDR set as 1%). Protein sequences used for identification originated from UniProt.

Mitochondria-targeted vehicle construction and medicine loading. Synthesis of MSN prepared by the two-phase method: 3.0 g of cetyltrimethylammonium bromide (CTAB) and 120 μL of triethanolamine (TEA) were dissolved in 60 mL of H_2O with stirring for 30 min, and then 12.0 mL of cyclohexane was added. Then, 3.0 mL of tetraethyl orthosilicate (TEOS) was added to react for 12 h with stirring. Finally, the product was collected by centrifugation and washed several times with water and ethanol. The final products were

dispersed in ethanol for further use.

Synthesis of MSN&PMO (MP) asymmetric nanoparticles: The MP asymmetric nanocomposites were prepared based on the anisotropic growth method: 5.0 mg of the above MSN nanoparticles was added to a solution containing 18.9 mL of water, 1.1 mL of ethanol and 30.0 mg of CTAB. After sonication for 30 min, 0.9 mL of ammonia was added under continuous stirring. After stirring for 30 min, 28 μL of 1,2-bis(triethoxysilyl)ethane (BTEE) was added to the reaction solution and the reaction was continued for 2 h. The MP asymmetric nanocomposites were collected by centrifugation and washed several times with ethanol. The samples were heated in an ethanol solution of NH_4NO_3 (6 g/L) at 60 °C for 4 h to remove the surfactant CTAB and then washed with deionized water three times.

Synthesis of TPP@MSN&PMO (TMP) asymmetric nanoparticles: 10.0 mg of the above MP asymmetric nanoparticles were added to a solution containing 50 mL of anhydrous ethanol, followed by 40 μL of (3-aminopropyl) triethoxysilane (APTES), which was stirred for 24 h at 80 °C, collected by centrifugation, and then washed with anhydrous ethanol three times and set aside. 60 mg of 1-(3-dimethylaminopropyl)-3-ethylcarbodiimide hydrochloride (EDC) and 80 mg of N-hydroxysuccinimide (NHS) were dissolved in 10.0 mL of PBS (pH 6.0). After adding TPP-COOH (2 mg), the mixture was allowed to react for 2 h at room temperature. Then, NH_2-MP asymmetric nanoparticles were added to the activated TPP solution. After stirring the reaction for 4 h, the TMP asymmetric nanoparticles were obtained by centrifugation and then washed three times with deionized water.

Synthesis of TMP-RES nanomedicine: The MP-RES nanomedicine was prepared by the physical absorption method: 5.0 mg of RES was dissolved in 5.0 mL of ethanol, and then 1.0 mg of MP nanoparticles were added to the solution, which was stirred for 24 h at room temperature, and centrifugation was used to collect the prepared MP-RES nanoparticles. The remaining RES molecules were washed away with ethanol. The loading of RES molecules was determined using HPLC based on their absorption peak area at 254 nm.

Physiochemical characterizations of nanoparticle. Scanning electron microscopy (SEM) images were captured by using field emission scanning electron microscopy (FESEM, Gemini 560, Germany). Transmission electron microscopy (TEM) and high-resolution transmission electron microscopy (HRTEM) observations were performed on JEM-2100F TEM with an accelerating voltage of 200 kV equipped with a post-column Gatan imaging filter (GIF-Tri-dium). High-resolution TEM (HRTEM), high-angle annular dark field imaging in the scanning TEM (HAADF-STEM), and energy-dispersive X-ray spectroscopy (EDS) mapping images were obtained on a JEM-2100F microscope (JEOL, Japan) with an accelerating voltage of 200 kV equipped with a postcolumn Gatan imaging

filter. The ultraviolet visible-near infrared (UV-Vis-NIR) spectra were recorded on a Shimadz spectrophotometer (UV-3150, Japan). The loading amount of RES in TMP was determined by high-performance liquid chromatography (HPLC, Agilent 1100). Nitrogen adsorption-desorption measurements were conducted to obtain information about the porosity. The measurements were conducted at 77 K with an ASAP 2420. Before measurements, the samples were degassed in a vacuum at 200 °C for at least 12 h. The Brunauer–Emmett–Teller (BET) method was utilized to calculate the specific surface areas, and the Barrett–Joyner–Halenda (BJH) model was utilized to calculate the pore volumes and the pore size distributions derived from the adsorption branches of isotherms. Fourier transform infrared (FTIR) spectra were recorded using a Fourier transform infrared spectrometer (ThermoFisher, Nicolet iS10, USA).

Animal experiments. 6-week-old C57BL/6 mice were placed in a hypoxia chamber exposed to IH for 8 hours each day, 38 days totally. The IH condition was set as 5 cycles per hour, keeping 5-7% oxygen for 30 s and 21% at peak. Every week, the mice received an intramuscular injection in the right hindlimb, with a total volume of 100 μL of the TMP-RES (1 μg/μL vehicle) administered across 10 evenly distributed injection sites. After 38 days, the mice were sacrificed and evaluated.

Statistical analysis. Data analyses were conducted by GraphPad Prism 9, expressed as the mean ± s.e.m. Analyses for fluorescence intensity of images were quantified by ImageJ. Statistic tests included two-tailed unpaired t test, Welch's t-test, and one-way ANOVA. P values were indicated as $*P<0.05$, $**P<0.01$, $***P<0.001$, considered as statistically significant.

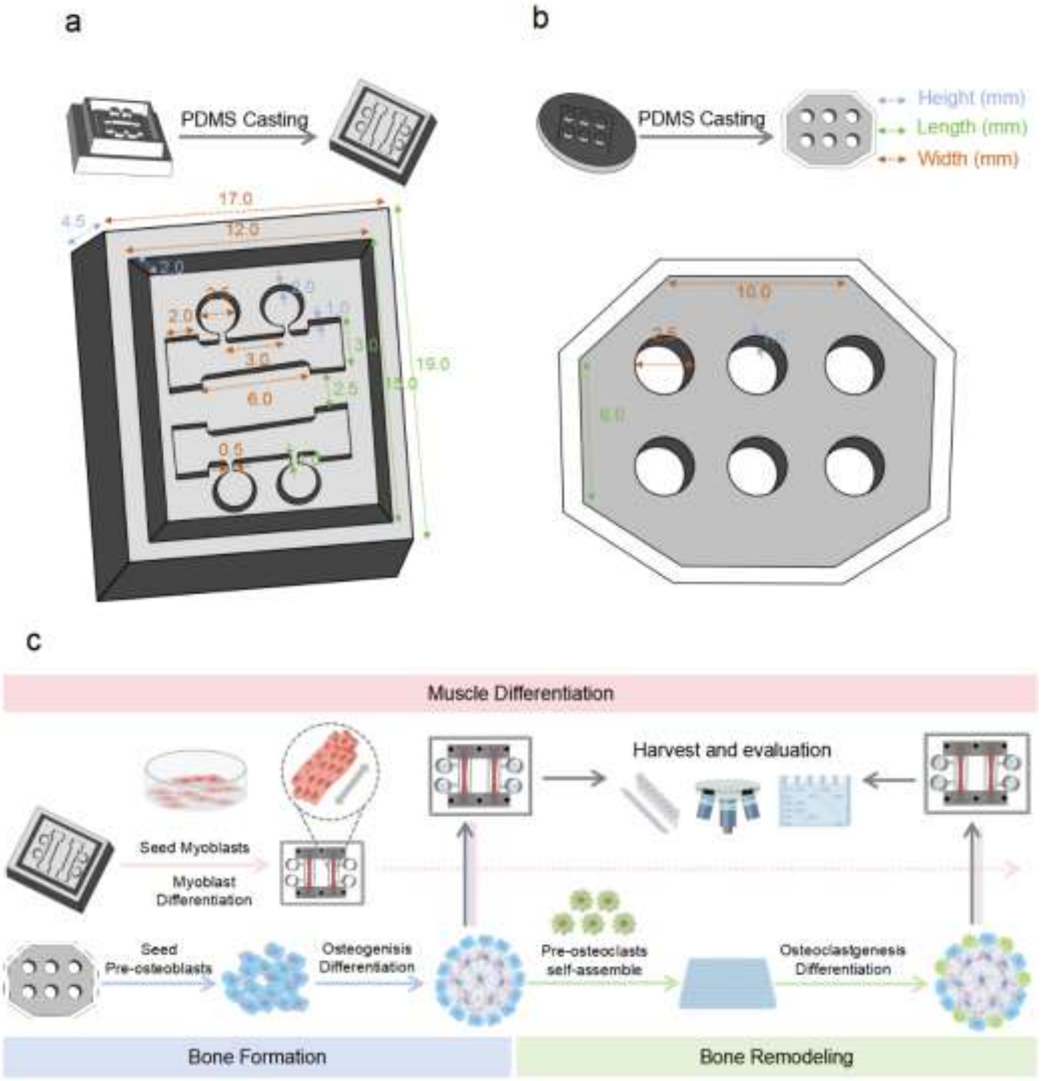

Figure S1. Size descriptions and protocols for MSK OoC fabrication. (a) Size description for MSK OoC. (b) Size description for chips used for BOs. (c) MOs and BOs coculture protocol for the MSK OoC.

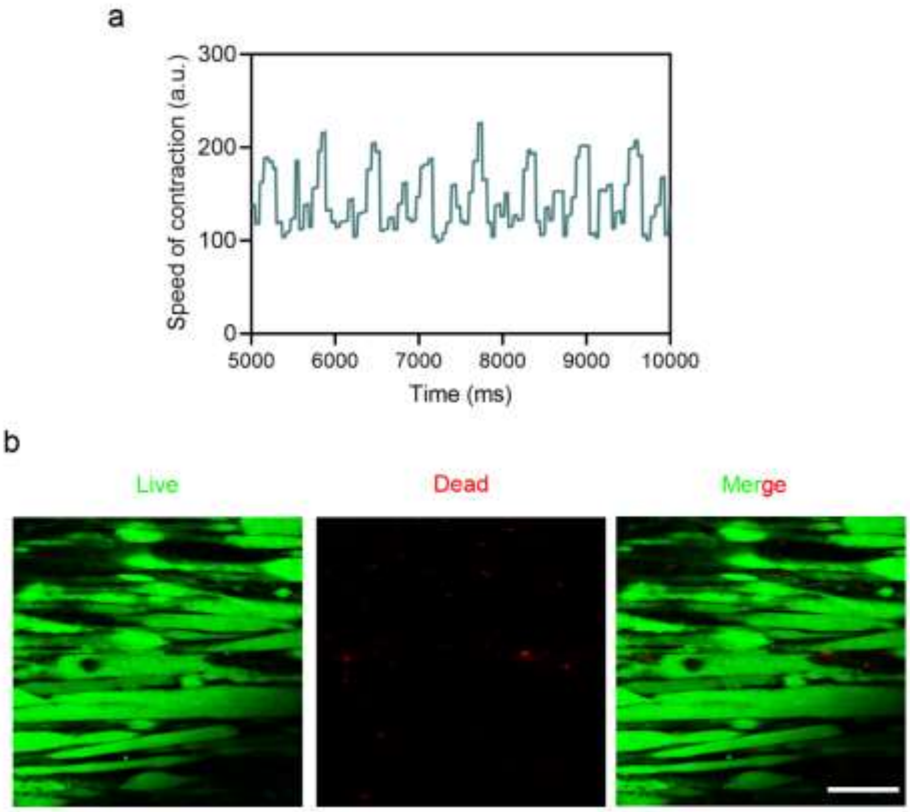

Figure S2. Characterizations of MOs. (a) Muscle contractility of MOs induced by electrical stimulation at frequency of 1 Hz. (b) Live/dead staining of 14-day MOs. Scale bar, 100 μm.

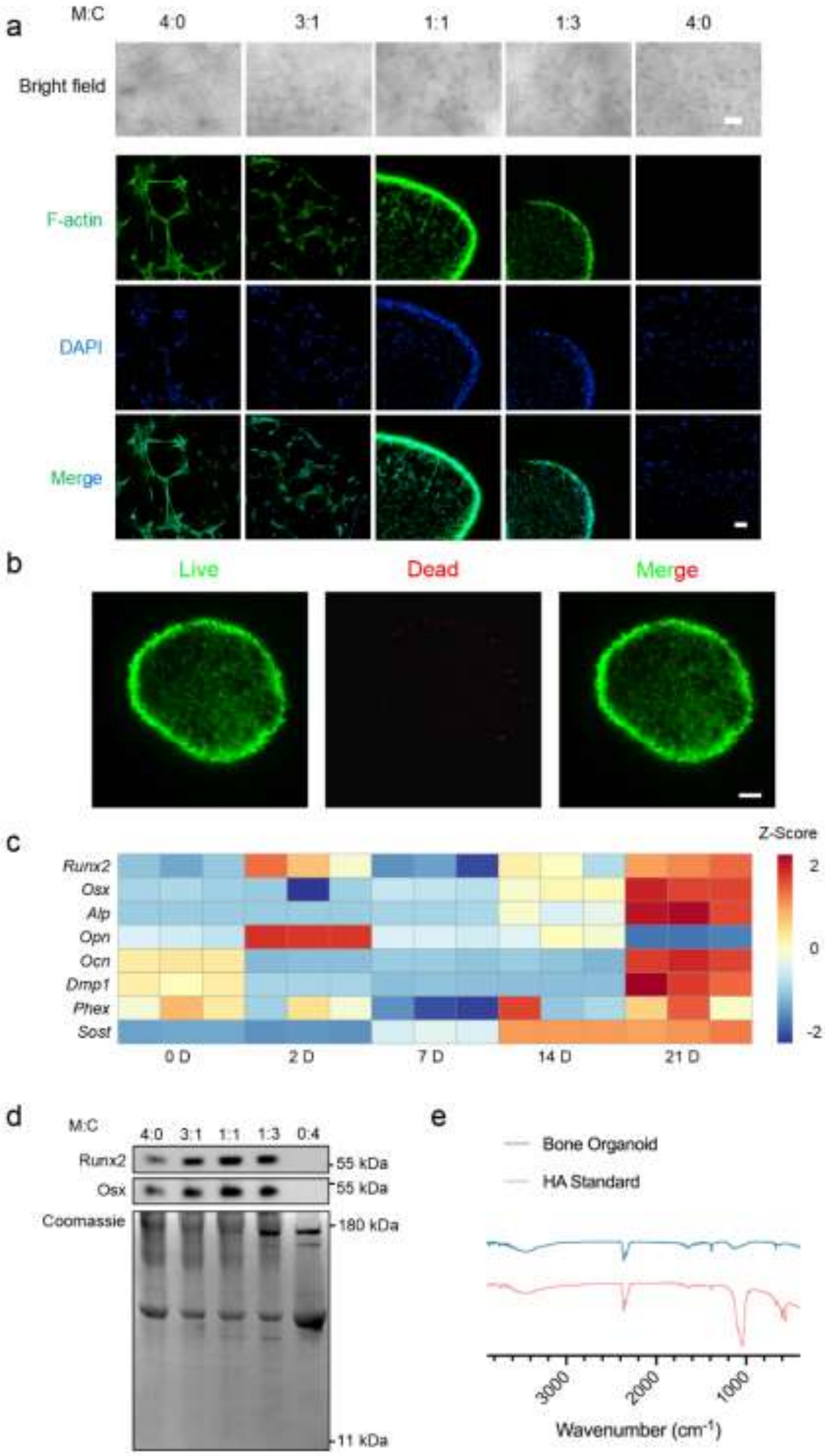

Figure S3. Construction and characterizations of BOs on the formation stage. (a) Bright-field pictures and F-actin staining. Scale bar, 100 μm. (b) Live/dead staining of 10-day BOs. Scale bar, 100 μm. (c) mRNA profile of osteogenic markers in BOs within 21 days. (d) Key osteogenic markers protein expression of BOs on the formation stage of various ratios of matrigel to collagen. (e) FTIR spectra of BOs and HA standard.

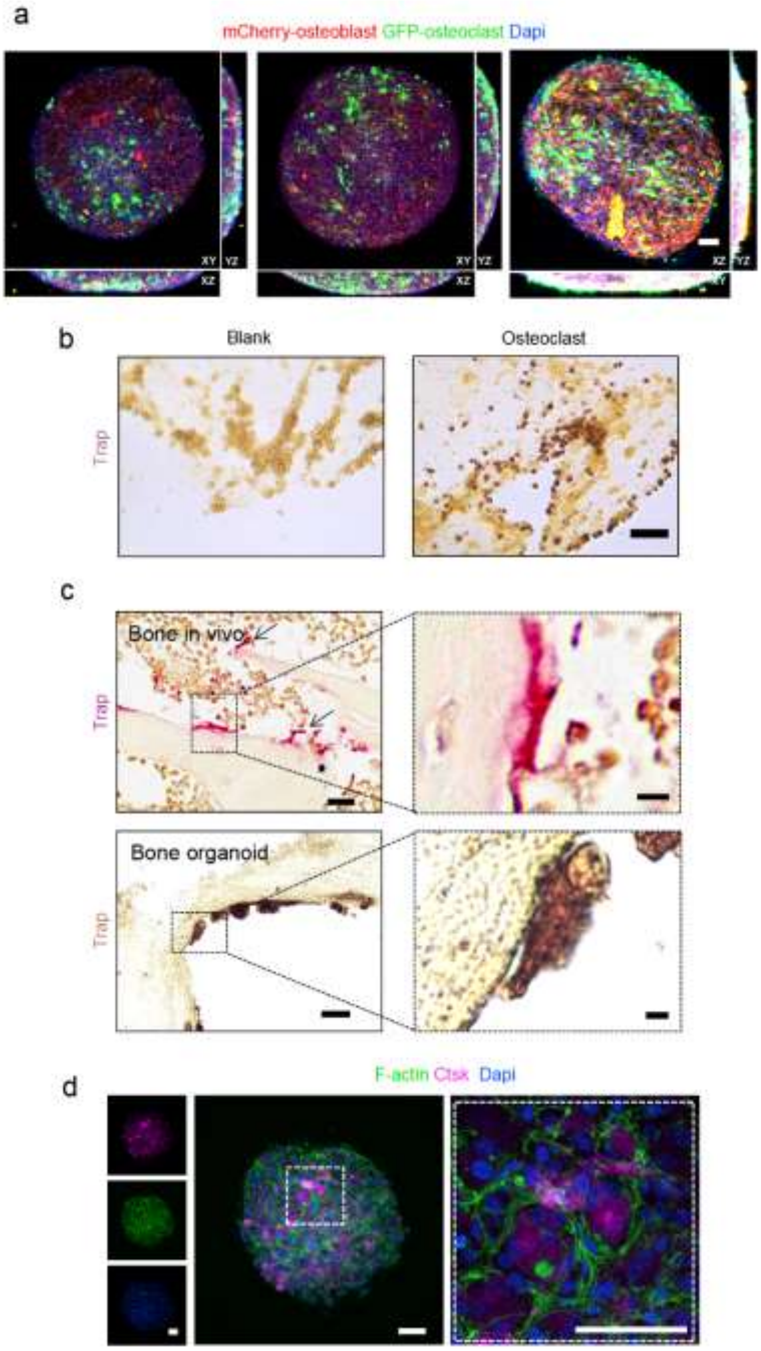

Figure S4. Construction and characterizations of BOs on the remodeling stage. (a) Different distributions of GFP-preosteoclast self-assemble onto BOs of different numbers, 1×10^4, 5×10^4, and 1×10^5 cells respectively. Scale bar, 100 μm. (b) Trap staining of osteoclasts after pre-osteoclasts self-assembling onto BOs. Scale bar, 100 μm. (c) Trap IHC staining of trabecular bone in vivo and a transverse slice of BOs. Scale bar, 20 μm (left), 5 μm (right). Arrow, Trap-positive osteoclast on the surface of bone in vivo; green dash line, pseudopods on the surface of BOs. (d) Representative confocal image of Ctsk staining with multinucleated fused giant cells on the remodeling BOs on the 49th day. Scale bar, 100 μm.

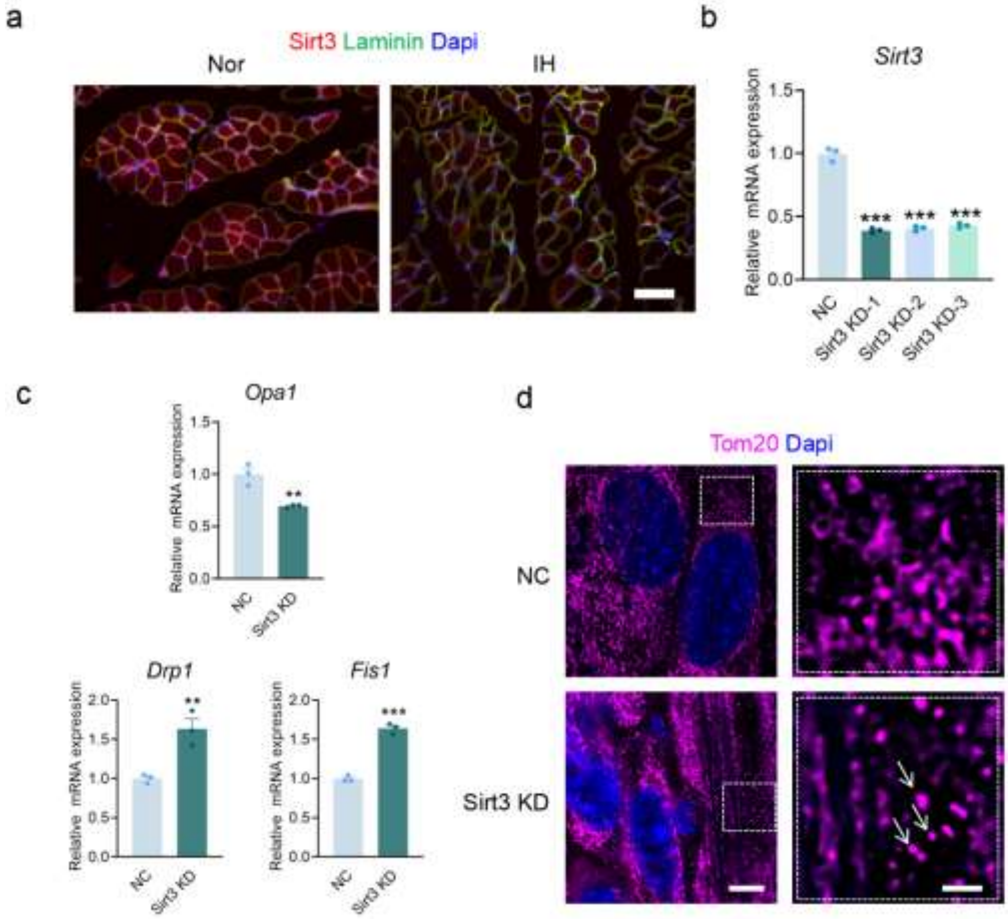

Figure S5. Sirt3 expression of muscle in vivo and characterizations of Sirt3 KD MOs. (a) Representative pictures of Sirt3 staining of genioglossus muscle of IH-treated mice. Scale bar, 50 μm. (b) KD efficiency of *Sirt3* by lentivirus using three different sequences. (c) *Opa1*, *Fis1*, and *Drp1* mRNA expression in Sirt3 KD MOs. (d) Mitochondria network of Sirt3 KD MOs. Scale bar, 5 μm (left), 1 μm (right). P values were calculated by one-way ANOVA (B) and two-tailed unpaired t test (c).

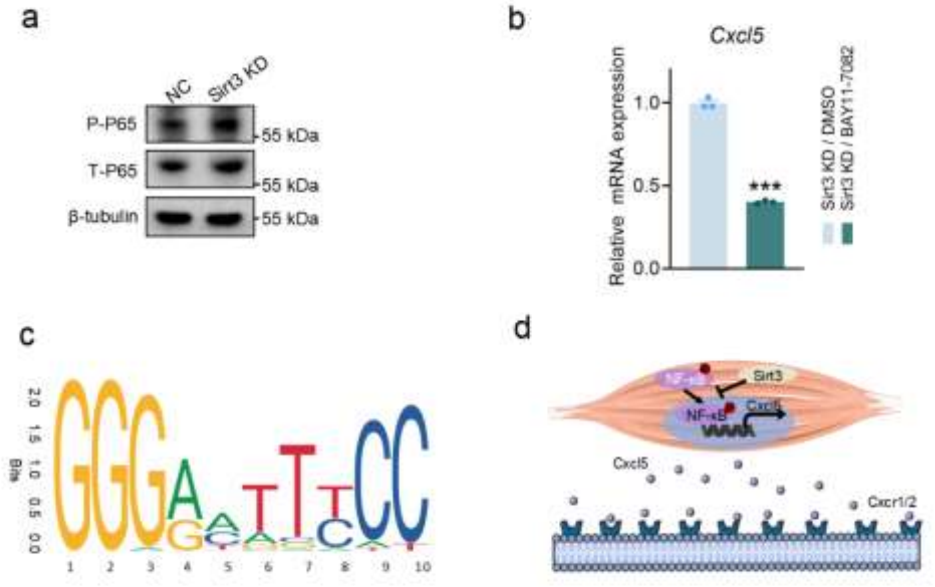

Figure S6. NF-κB phosphorylation-induced Cxcl5 transcription in Sirt3 KD MOs. (a) WB of P65 phosphorylation (P-P65) in Sirt3 KD MOs (T-P65: total P65). (b) mRNA expression of *Cxcl5* after NF-κB inhibitor treatment (BAY 11-7082). (c) Prediction of a sequence of core binding sequence location in the *Cxcl5* promotor. (d) Mechanism diagram for Sirt3-NF-κB-Cxcl5 pathway. ns: not significant; *P< 0.05, **P< 0.01, ***P< 0.001; mean ± s.e.m., n≥ 3. P values were calculated by two-tailed unpaired t test.

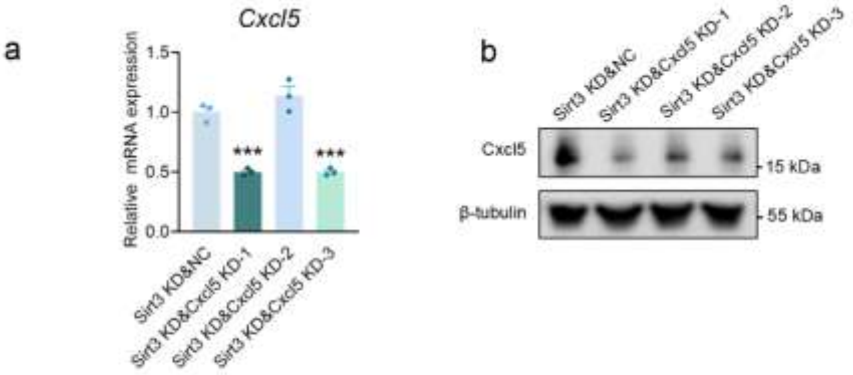

Figure S7. Cxcl5 KD efficiency based on Sirt3 KD cell line. (a) mRNA and (b) protein expression in Sirt 3 KD cell by lentivirus using three different sequences. ns: not significant; *P< 0.05, **P< 0.01, ***P< 0.001; mean ± s.e.m., n≥ 3. P values were calculated by one-way ANOVA.

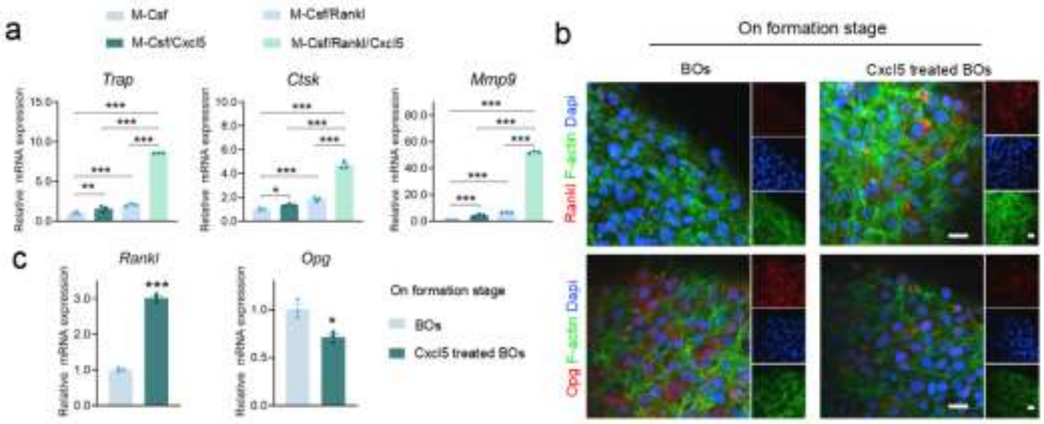

Figure S8. Cxcl5 regulation of osteoblast-osteoclast interaction through Rankl/Opg. (a) mRNA expression of *Trap*, *Ctsk*, and *Mmp-9* of osteoclasts in 2D cultured with or without Cxcl5 or Rankl. (b) Representative confocal images and (c) mRNA expression of *Rankl* and *Opg* of BOs on the formation stage. Scale bar, 20 μm. ns: not significant; $*P < 0.05$, $**P < 0.01$, $***P < 0.001$; mean ± s.e.m., $n \geq 3$. P values were calculated by one-way ANOVA (a) and two-tailed unpaired t test (c).

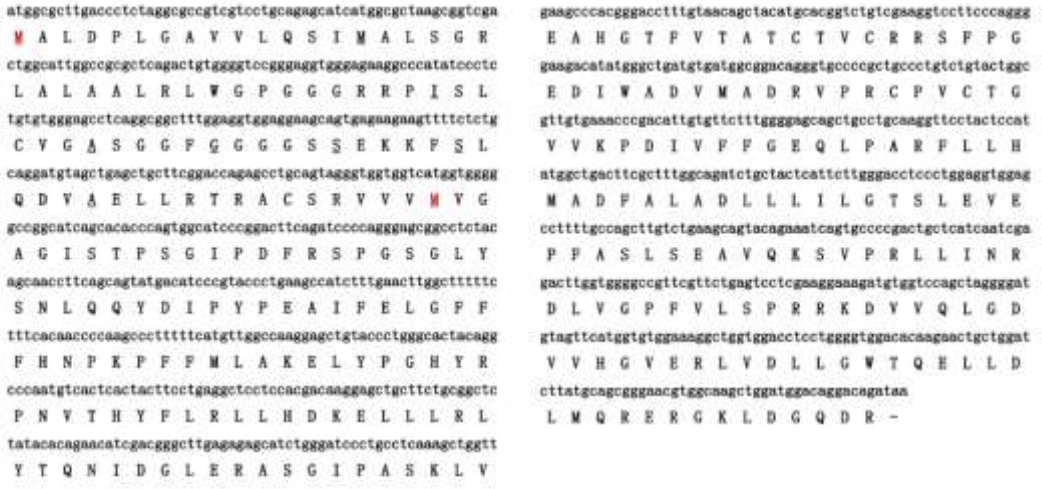

Figure S9. Sirt3 OE and MTS-Sirt3 OE sequences (two corresponding initiator codons are marked in red).

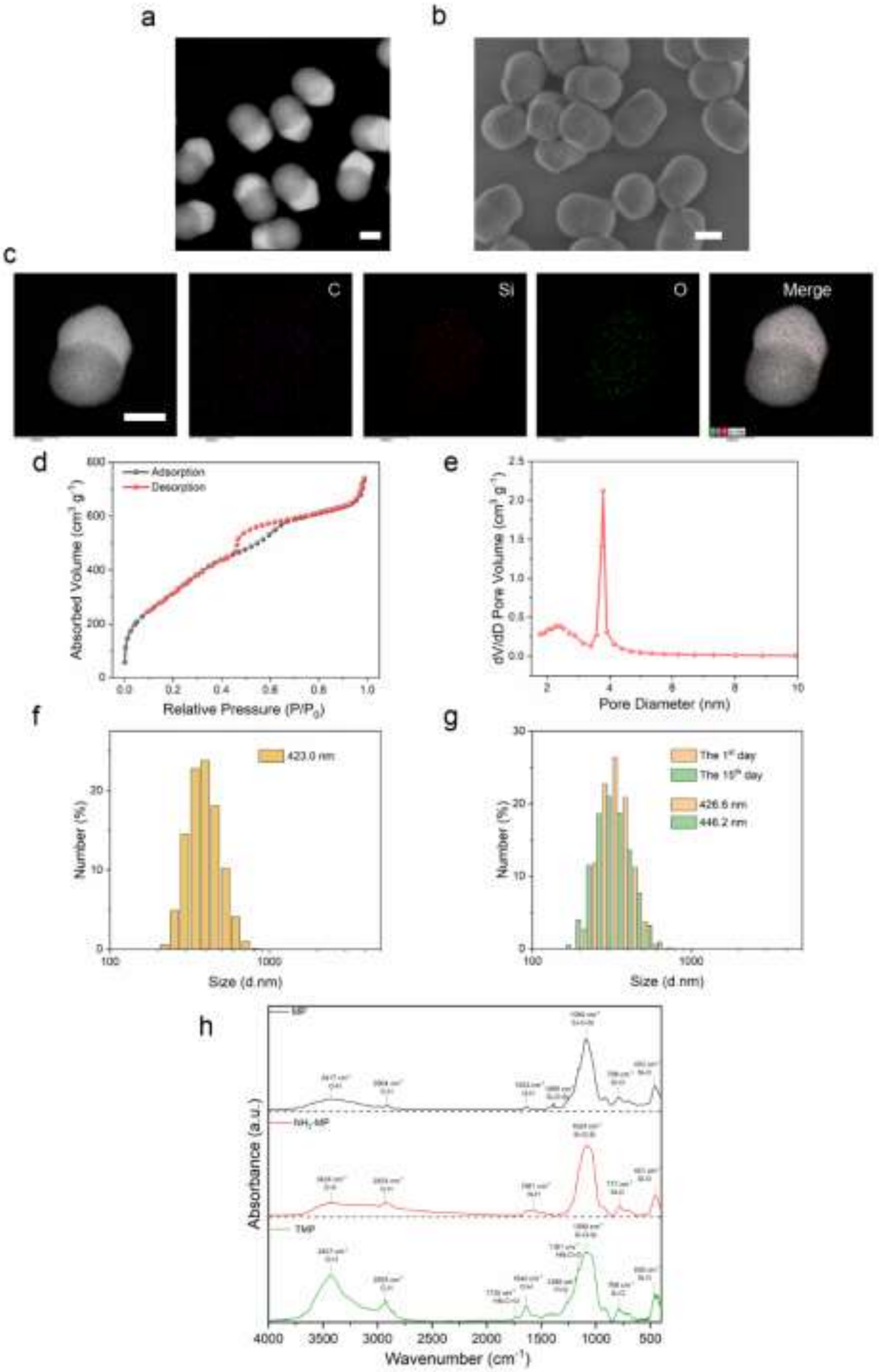

Figure S10. Characterizations of the asymmetric-structured mesoporous. (a) High-angle annular dark-field scanning TEM, (b) SEM, (c) EDS, (d) BET surface area, (e) and pore size distribution of MP. Scale bar, 100 nm. (f) Hydrodynamic diameter of TMP-RES in the medium. (g) Hydrodynamic diameter of TMP-RES kept in the medium for 15 days. (h) FTIR spectra of the MSN&PMO (MP), NH_2-MSN&PMO (NH_2-MP), TPP@MSN&PMO (TMP).

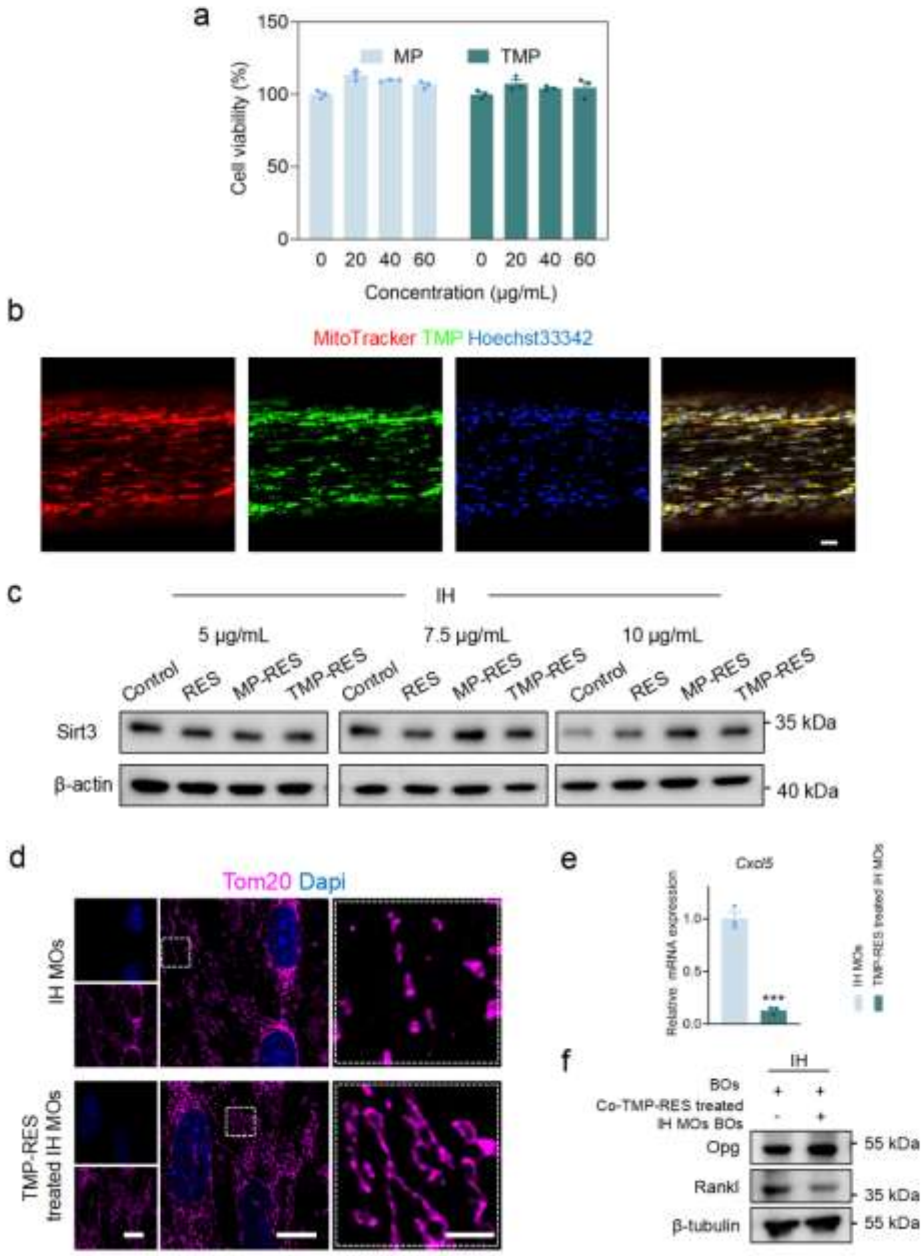

Figure S11. Biological characterizations of the nanomedicine. (a) CCK8 test of MP and TMP to C2C12 cell. (b) Mitochondrial targeting efficiency of TMP inside the MOs. Scale bar, 40 μm. (c) Sirt3 expression of Nanomedicine dose selection. (d) Representative confocal images showing mitochondria net integrity in MOs after TMP-RES treatments under IH. Scale bar, 10 μm (left, middle), 2 μm (right). (e) Relative mRNA expression of *Cxcl5* of MOs after TMP-RES treatments under IH. (f) Rankl and Opg expression of BOs cocultured with targeted treatment of MOs under IH. ns: not significant; $^{*}P< 0.05$, $^{**}P< 0.01$, $^{***}P< 0.001$; mean ± s.e.m., n≥ 3. P values were calculated by two-tailed unpaired t test.

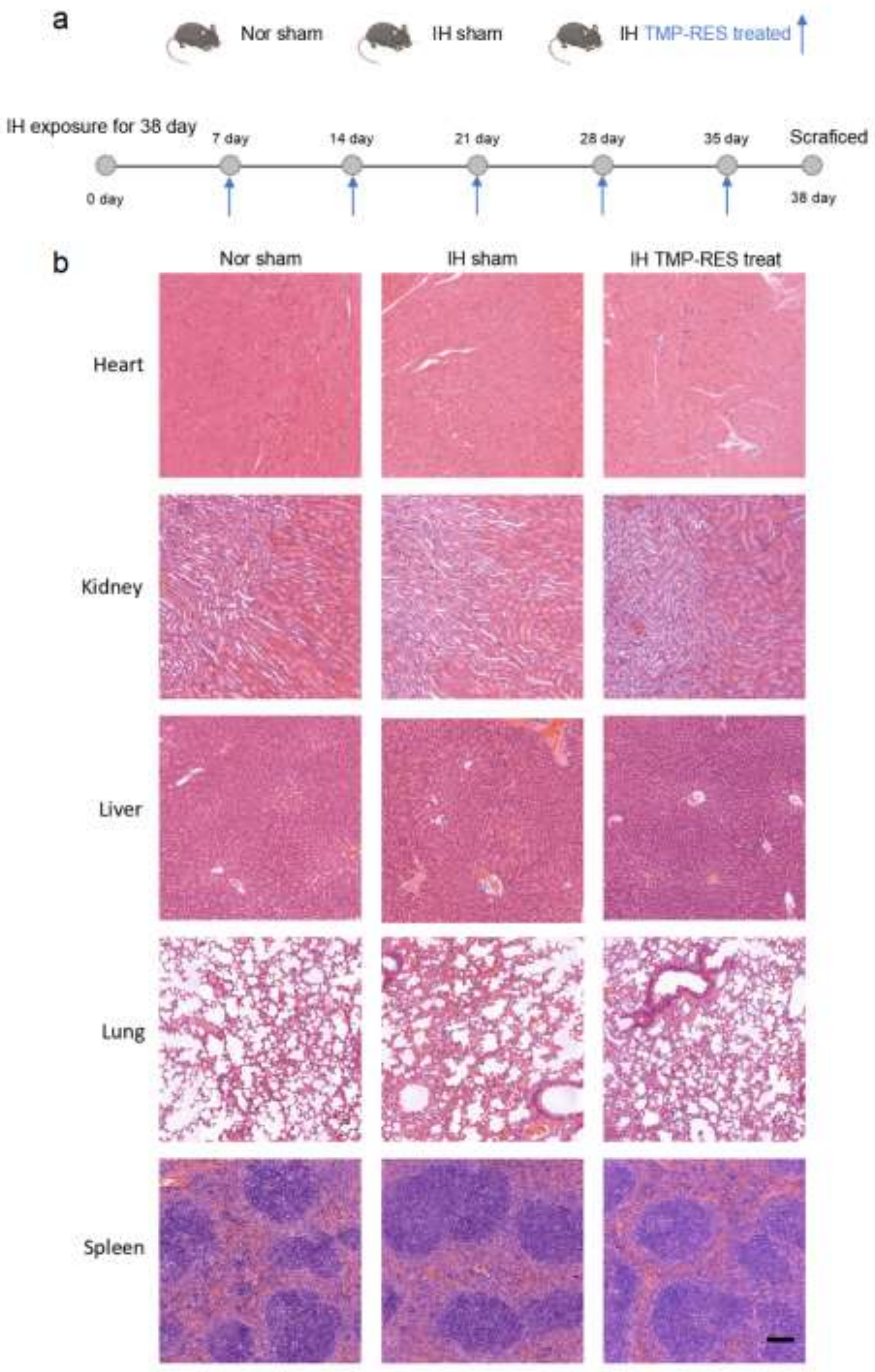

Figure S12. In vivo safety assessment of nanomedicine. (a) Schematic diagram for animal experiment. (b) HE staining of heart, kidney, liver, lung, spleen. Scale bar, 100 μm.

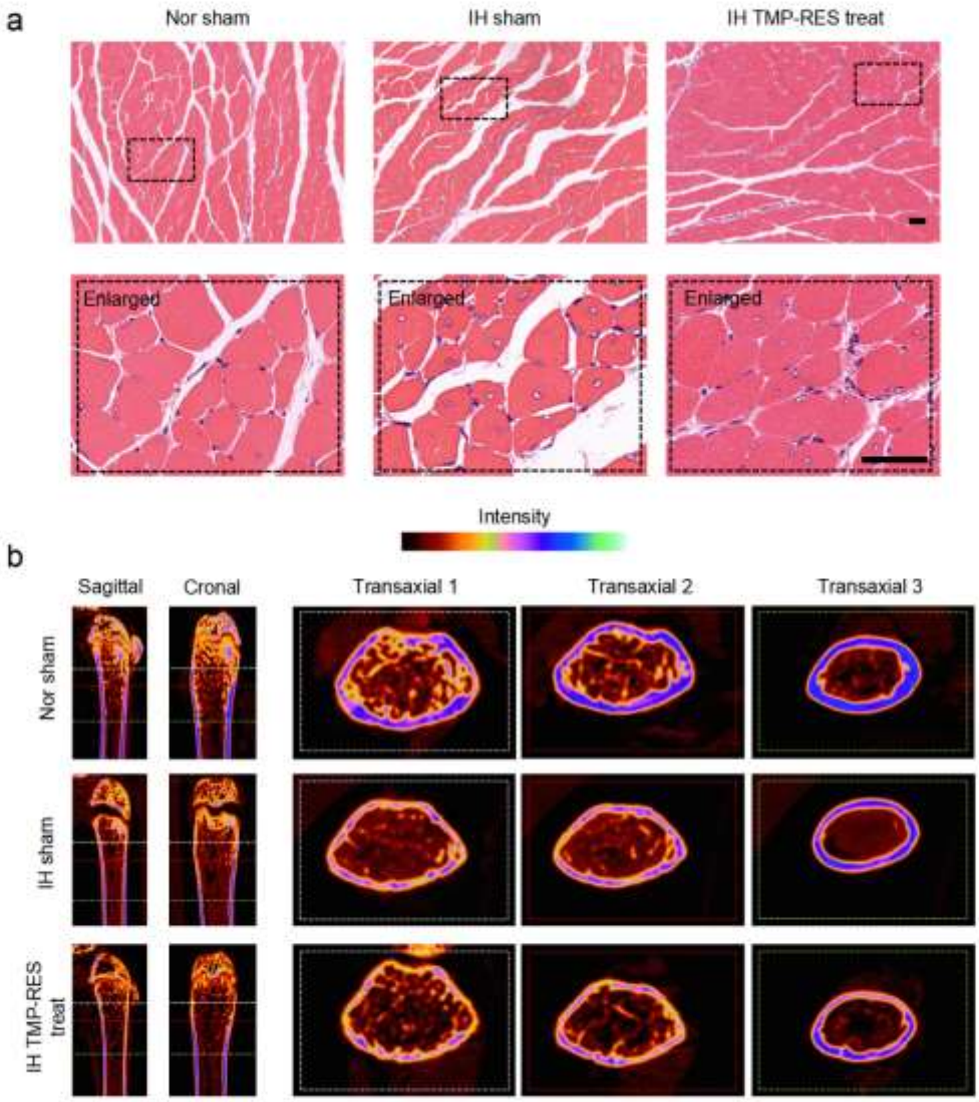

Figure S13. Evaluation of the therapeutic effectiveness of nanomedicine on muscle-bone hypoxic injury in vivo. (a) Centralized nuclei increased in the gastrocnemius muscle after chronic IH exposure, while a decreasing trend was observed after nanomedicine treatment in the representative HE staining. Scale bar, 50 μm. (b) Nanomedicine administration via intramuscular injection ameliorated IH-induced osteoporosis in the representative micro-CT images from the sagittal, coronal, and three cross-sectional planes of distal femur.

Table. 1 RT-qPCR Primers

Primer name	Sequence 5' to 3'
Runx2 forward primer	GCCAGGCAGGTGCTTCAGAACT
Runx2 reverse primer	CTGGGCGGGGTGTAGGTAAAG
Osx forward primer	GGAAAGGAGGCACAAAGAAGC
Osx reverse primer	CCCCTTAGGCACTAGGAGC
Alp forward primer	CCAACTCTTTTGTGCCAGAGA
Alp reverse primer	GGCTACATTGGTGTTGAGCTTTT
Opn forward primer	CCCGGTGAAAGTGACTGATT
Opn reverse primer	GGCTTTCATTGGAATTGCTT
Ocn forward primer	GCCCAGACCTAGCAGACAC
Ocn reverse primer	TGGGCTTGGCATCTGTGAG
Dmp1 forward primer	CACGGACAGCAGTGAATCTGG
Dmp1 reverse primer	GCCGGTCCCCGTACTCTTA
Sost forward primer	AGCCTTCAGGAATGATGCCAC
Sost reverse primer	CTTTGGCGTCATAGGGATGGT
Phex forward primer	CCAACCGAGGCATTCGGATT
Phex reverse primer	AGCTTGGAAACTTAGGAGACCT
Opg forward primer	ACCCAGAAACTGGTCATCAGC
Opg reverse primer	CTGCAATACACACACTCATCACT
RankL forward primer	CAGCATCGCTCTGTTCCTGTA
RankL reverse primer	CTGCGTTTTCATGGAGTCTCA
Drp1 forward primer	ACCGGGAATGACCAAAGTACC
Drp1 reverse primer	TGGGATTACTGATGAACCGAAGA
Fis1 forward primer	AGAGCACGCAATTTGAATATGCC
Fis1 reverse primer	ATAGTCCCGCTGTTCCTCTTT
Opa1 forward primer	TGGAAAATGGTTCGAGAGTCAG
Opa1 reverse primer	CATTCCGTCTCTAGGTTAAAGCG
Sirt1 forward primer	TGATTGGCACCGATCCTCG
Sirt1 reverse primer	CCACAGCGTCATATCATCCAG
Sirt2 forward primer	GCGGGTATCCCTGACTTCC
Sirt2 reverse primer	CGTGTCTATGTTCTGCGTGTAG
Sirt3 forward primer	GGCTCTATACACAGAACATCGAC
Sirt3 reverse primer	TAGCTGTTACAAAGGTCCCGT
Sirt4 forward primer	CAAGAAACTCCTCGTGATGACA
Sirt4 reverse primer	GTCAGTGCGGGCGTAAAGT
Sirt5 forward primer	AATATGGCAGACTTTCGGAAGTG
Sirt5 reverse primer	ACACCTGTGATGGGTTTCGAG
Sirt6 forward primer	CCTGGTCAGCCAGAACGTAG
Sirt6 reverse primer	TACTGCGTCTTACACTTGGGA
Sirt7 forward primer	GCACTTGGTTGTCTACACGG

Sirt7 reverse primer	TGTCCATACTCCATTAGGACCC
Myh1 forward primer	GGAGTCAGGTGAATACTCACG
Myh1 reverse primer	GCATGAGCTAAGGCACTCTTG
Myh2 forward primer	ACTTTGGCACTACGGGGAAAC
Myh2 reverse primer	CAGCAGCATTTCGATCAGCTC
Myh7 forward primer	GACTGTCAACACTAAGAGGGTC
Myh7 reverse primer	GCCCCAAAATGGATTCGGATG
Casq1 forward primer	TGGAGGACTGGCTGGAGGAC
Casq1 reverse primer	GTCGTCGTCATCATCATCGTCATC
Pmca forward primer	CAGCACAGTCTCAGAGCAACG
Pmca reverse primer	GCTACGATGGTGGAGAAGTTGTC
Serca1 forward primer	AGTTCATCCGCTACCTCATCTCC
Serca1 reverse primer	GCCCATCAGTCACCAAGTTCAC
Hlf forward primer	CCGGCTTCAGCACTATGAAC
Hlf reverse primer	ACGAATGACAATCCTTGTGTCTC
Fras1 forward primer	GCTTGTCTGTATCAGGGCTCC
Fras1 reverse primer	CTTCTCCCTTCTCAAAGGCAC
Heg1 forward primer	TTCTCAAACAGAGAGTGGGACA
Heg1 reverse primer	TTCCTGGCCGGATGAATTTCT
Cxcl5 forward primer	TCCAGCTCGCCATTCATGC
Cxcl5 reverse primer	TTGCGGCTATGACTGAGGAAG
Xkr5 forward primer	CAGGCCACCATACCATGCTAT
Xkr5 reverse primer	CACACGGTAAACTCGGCAGA
18s forward primer	GTAACCCGTTGAACCCCATT
18s reverse primer	CCATCCAATCGGTAGTAGCG
